# Immunogenicity and Efficacy Evaluation of Subunit Astrovirus Vaccines

**DOI:** 10.3390/vaccines7030079

**Published:** 2019-08-02

**Authors:** Mehdi R.M. Bidokhti, Karin Ullman, Anne Sofie Hammer, Trine Hammer Jensen, Mariann Chriél, Siddappa N. Byrareddy, Claudia Baule

**Affiliations:** 1 Department of Virology, Immunobiology and Parasitology, The National Veterinary Institute (SVA), SE-751 89 Uppsala, Sweden; 2 Department of Pharmacology and Experimental Neuroscience, College of Medicine, University of Nebraska Medical Center (UNMC), Omaha, NE 68198-5800, USA; 3 The National Veterinary Institute, Technical University of Denmark (DTU), DK-8200 Aarhus N, Denmark

**Keywords:** astrovirus, capsid protein, subunit vaccine, immunogenicity, infection

## Abstract

A full understanding of the immune response to astrovirus (AstV) infection is required to treat and control AstV-induced gastroenteritis. Relative contributions of each arm of the immune system in restricting AstV infection remain unknown. In this study, two novel subunit AstV vaccines derived from capsid protein (CP) of mink AstV (MAstV) such as CPΔN (spanning amino acids 161–775) and CPΔC (spanning amino acids 1–621) were evaluated. Their immunogenicity and cytokine production in mice, as well as protective efficacy in mink litters via maternal immunization, were studied. Truncated CPs induced higher levels of serum anti-CP antibodies than CP, with the highest level for CPΔN. No seronegativity was detected after booster immunization with either AstV CP truncates in both mice and mink. All mink moms stayed seropositive during the entire 104-day study. Furthermore, lymphoproliferation responses and Th1/Th2 cytokine induction of mice splenocytes ex vivo re-stimulated by truncated CPs were significantly higher than those by CP, with the highest level for CPΔN. Immunization of mink moms with truncated CPs could suppress virus shedding and clinical signs in their litters during a 51-day study after challenge with a heterogeneous MAstV strain. Collectively, AstV truncated CPs exhibit better parameters for protection than full-length CP.

## 1. Introduction

Immunization against the viral pathogens causing gastrointestinal disorders is one of the most important concerns in public and animal health. Development of viral vaccines has traditionally been based on the use of attenuated live or inactivated viruses to induce protective immunity. This strategy has generated both live attenuated and inactivated vaccines currently in use for different diseases. Despite its efficacy, it has been difficult to extend this strategy to many viral pathogens because attenuated strains are either difficult to produce or unreliable, and posing safety risks. Inactivated vaccines, on the other hand, are safe but poorly immunogenic [1,2]. As a result, alternative vaccine development strategies are being investigated and tested, such as recombinant DNA [3], protein-based subunit vaccines [4], viral vector-based vaccines [5], vaccine generation by reverse genetics and codon optimization approaches [6], and more recently mRNA vaccines [7]. Several efforts are aimed to increase the immunogenicity and the safety of vaccines. In particular, subunit vaccines target specific epitopes recognized for immunity and are safe since they do not involve replication of the viral pathogen. However, these need to be combined with adjuvants to stimulate good immune responses and often require repeated immunization. Vital aspects for subunit vaccines effectivity are that the right immunogens for protection are incorporated in the design, and that the vaccine stimulates the humoral and, more importantly, the cellular arm of the immune response [4]. Therefore, the development of vaccines based on recombinant proteins require strategies that target a strong Th1/Th2 immunity, which is vital for protection and resistance to infections.

Astroviruses (AstVs) are positive-sense, single-stranded RNA, non-enveloped viruses that have an icosahedral capsid, small size (28–30 nanometers in diameter), and star-like appearance in electron microscopy (EM) [8]. The length of the genome is around 6.6 kb, and it contains three open reading frames (ORFs). ORF1a and ORF1b encode precursors of the viral nonstructural proteins, while ORF2 encodes for the precursor of the viral structural protein [9,10,11]. It has been shown that the subgenomic RNA of human astrovirus (HAstV), derived from ORF2, encodes a single, large viral structural capsid protein (CP) [12]. This capsid polyprotein precursor contains around 775–785 amino acid (aa) residues, depending on the virus strain, and has a molecular mass of 87–90 kilodalton (kDa) [13,14]. The AstV CP is an external structural barrier that not only encapsidates nucleic acids but also interacts with the host to define cell tropism, mediates cell entry, and triggers the host immune response [15]. As a non-enveloped virus, AstV exhibits an intriguing feature in that its maturation requires extensive proteolytic processing of the AstV CP both inside and outside the host cell [16].

AstV infection, as the second common cause of gastroenteritis after rotaviruses, begins by binding to an unidentified receptor(s) on epithelial cells after fecal-oral transmission followed by entry via endosomes [17]. In animals, AstVs cause gastrointestinal disease in mink [8], cattle [18], sheep [19], pig [20], cats [21], dogs [22], and marine mammals [23]. In turkey and chicken, it also causes nephritis, the runting-stunting syndrome, and gastrointestinal disease [24,25]. In addition, both classic and novel AstVs were identified as the cause of unexpected central nervous system (CNS) infections in human [26,27] and in animals such as mink [28], sheep [29], and cattle [30], highlighting that these viruses may bypass the gastrointestinal tract and infect the brain and other organs.

Mink astrovirus (MAstV) is the causative agent of pre-weaning diarrhea syndrome in young mink litters [8]. The syndrome is referred to “sticky”, “greasy”, or “wet” litters, and is characterized by diarrhea and excessive secretion from cervical apocrine glands in mink litters usually at the age of 1–4 weeks [8,11]. This results in the soiling of the neck and back, and a wavy appearance of fur. Post mortem examination of litters dying from this syndrome reveals non-specific catarrhal enteritis with hydropic epithelial cell degeneration, infiltration of mononuclear cells in the villous propria, and hypersecretion of the apocrine neck glands [31]. The economic losses in Scandinavia alone amount to 750 million Danish kroners (equal to 124 million US dollars), distributed in terms of mortality, losses due to bad skin quality, associated labor, and costs for antibiotic treatment due to the misuse of antibiotics, which also impacts on antibiotic resistance [32]. Thus, there is an urgent need for a vaccine that protects mink against the disease and reduces economic losses to the mink farming and industry. Due to difficulties vaccinating mink litters, eliciting high levels of maternal IgG antibodies, transferred to litters upon vaccination of female adult minks, has been highlighted as a main criterion for such a vaccine candidate. However, for human or birds, cellular and mucosal immunities could also be considered for protection beside humoral immune response upon direct vaccination.

The difficulty to grow AstVs from different species has limited the possibility to produce conventional vaccines based on live attenuated virus [33]. Therefore, molecular biology-based methods are the current alternative to engineer subunit vaccines based on recombinant proteins or oligonucleotides. The HAstV CP that contains the immunogenic domains has been expressed mostly for functional studies [16,34,35]. Our previous work with a full-length CP of MAstV expressed in bacteria has shown partial protection against the manifestation of clinical symptoms of pre-weaning diarrhea syndrome in mink [36]. In an immunization trial with a full-length CP of the chicken AstV expressed in baculovirus, partial protection was also reported [37]. In the present study, we compared the immune responses to full-length, and N- and C-terminally truncated forms of MAstV CP. The antibody response, proliferative ability, and induction of cytokines in splenocytes were determined following immunization of mice. We also evaluated maternal passive immunization in minks by investigating virus shedding and clinical signs in litters after challenging with a heterologous MAstV strain.

## 2. Materials and Methods

### 2.1. Construction and Expression of MAstV Vaccine Candidates

The construction and expression of the full-length and truncated MAstV CP of strain DK5790 using pDual-GC vector (Agilent Technologies, Santa Clara, CA, USA) have been described [36]. CP refers to the full-length capsid protein (spanning amino acids (aa) 1–775 of CP); CPΔN refers to an N-terminal truncated protein (spanning aa 161–775 of CP); CPΔC is a C-terminal truncated protein (spanning aa 1–621 of CP). The MAstV CPs were expressed in stably transfected fetal mink cells and were purified by affinity in a nickel resin, as described in details in our previous study [36].

### 2.2. Immunogenicity Evaluation in Mice

Four groups, each containing eight Naval Medical Research Institute (NMRI) mice at the age of 4 weeks (Charles River Laboratories, Wilmington, MA, USA) were used in this study. The mice trial (project number: 10/122) were carried out at the National Veterinary Institute of Sweden in accordance with both institutional and Swedish National Committee for the protection of animals used for scientific purposes’ guidelines (Ethical number: C236/8). Three groups were injected subcutaneously with 0.2 mL of a mixture of 5 µg of CP-, CPΔN-, or CPΔC-proteins in phosphate-buffered saline (PBS) with 10 µg of AbISCO-100 adjuvant (Isconova, Uppsala, Sweden) per mouse. Mice in the sham group (*n* = 8) were also injected with 0.2 mL of PBS containing 10 µg of AbISCO-100 adjuvant. Three weeks after the first immunization, the mice received a second injection of AstV protein or control cell lysate applied as before. Blood sera were collected three weeks after each immunization and stored at −20 °C. We used an indirect enzyme-linked immunosorbent assay (ELISA) to measure humoral immune response. Animals in each group were sacrificed three weeks after the second immunization. Splenocytes were harvested, seeded in 96-well plates and re-stimulated with various concentrations of the purified AstV CPs in order to measure proliferation activity and cytokine secretions to study cellular immune responses.

### 2.3. Protective Efficacy Evaluation in Mink

Seventeen adult female wild type minks at the age of 1-year old were purchased from a MAstV-free farm in Denmark and transported to the National Veterinary Institute, Technical University of Denmark, where all mink experiments were carried out in accordance with both institutional and Danish Animal Care and Ethics Committee’s national guidelines (Ethical number: 08/561-1534). All minks were first tested for antibodies to MAstV, with negative results. Their polymerase chain reaction (PCR) results also did not show any active AstV infection. The adult female minks then were injected subcutaneously with 100 µg of CPΔN (*n* = 6), CPΔC (*n* = 6), or with pDual-GC-vector-transfected cell lysate as sham (*n* = 5) combined with an equal amount of Freund’s adjuvant (Sigma-Aldrich, St. Louis, MO, USA) (Table 1). The immunization was repeated three weeks after. The 1-day-old litters (*n* = 89) born to these immunized female moms were inoculated orally with a high dose of challenge MAstV Danish strain DK7627 (10^7^ virus genome copies). The author veterinarians recorded clinical symptoms in the litters on a daily basis. Totally, 414 fecal samples were collected at different days post-challenge throughout the observation period (51 days) and tested with a quantitative real-time RT-PCR (targeted non-structural gene of MAstV, Threshold = 0.02, unpublished data) and one-step RT-PCR kits (Qiagen, Hilden, Germany) for determination of virus shedding after challenge (Table 1).

**Figure 1:**
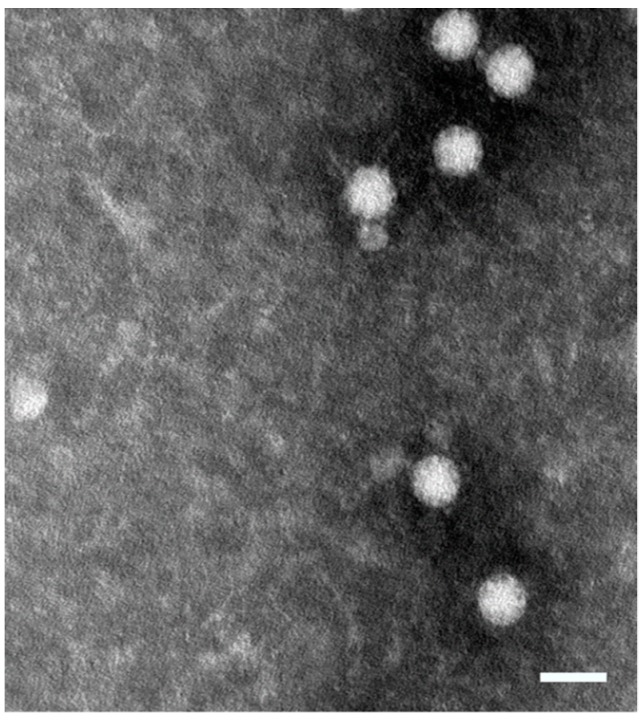
Electron microscopy (EM) graph of mink astrovirus (MAstV), Danish strain DK7627 used for challenge experiment of litters. To prepare the infectious material for the experiment, fecal samples of a naturally-infected mink were examined by EM and small star-like particles of MAstV, 30 nm in diameter, were observed. Further analyses with a MAstV-specific PCR and sequencing also detected the genome of the MAstV Danish strain DK7627 in the sample. The number of RNA copies of MAstV in this infectious material measured by a quantitative PCR was 10^7^/mL. This was later used for the challenge of litters. The white bar scale is 50 nm. The EM examination was performed at the Swedish Institute for Infectious Disease Control (SMI).

The challenge materials came from a farm that reported extensive MAstV infection and suffering from shaking mink syndrome. To prepare the challenge materials, the fecal samples were first tested for positive MAstV by a quantitative real-time PCR [36] and also EM (Figure 1). Sequencing results showed the presence of a heterogeneous Danish strain DK7627 in the fecal samples compared to the strain DK5790 that was used to design and prepare AstV CP subunit vaccine candidates. Using 0.2 µm filters, the positive fecal samples were filtered to avoid bacterial and fungal contamination. These filtered materials were further exploited as MAstV-positive fecal filtrate for challenging the litters born to immunized moms during this experimental study.

### 2.4. Indirect ELISA

To detect the anti-MAstV CP specific antibodies in serum samples of mice and minks, an indirect ELISA was developed and used as previously described [36]. Briefly, a recombinant MAstV CP was first expressed in BL-21 competent *Escherichia coli* cells, then lysed mechanically by sonication and purified using HisTrap™ columns (GE Healthcare, Uppsala, Sweden) in nickel affinity chromatography. The wells of high-binding ELISA plates (Nunc-Immuno™ Plates, Thermo Fisher Scientific, Waltham, MA, USA) were then coated with 100 ng/well of the purified recombinant MAstV CP diluted in carbonate buffer (pH 9.6, Sigma Aldrich, St. Louis, MO, USA) and incubated overnight at 4 °C. Next, the plates were washed three times with PBS-T (PBS with 0.05% Tween 20, Thermo Fisher Scientific, Waltham, MA, USA) and blocked with 100 µL/well of blocking solution (PBS-T containing 5% of skimmed milk) for 1 h. The plates were washed with PBS-T three times. One hundred microliters of serum samples diluted 1:100 in blocking buffer were added to each well, and the plates were incubated at 37 °C for 1 h. After washing with PBS-T, horseradish peroxidase-labeled mouse anti- mustelid IgG secondary antibody (MyBioSource, San Diego, CA, USA) diluted 1:1600 in blocking buffer was added, and the plates were incubated for another 1 h. After three washes as before, the substrate solution (tetramethylbenzidine) was added; the reaction was stopped by adding 100 µL 1M sulfuric acid (H_2_SO_4_) to each well. All incubation steps were performed at room temperature. The optical density (OD) was measured at 450 nm in an ELISA microplate reader (Biocompare, San Francisco, CA, USA). The experiment was done in duplicate. The mean OD for the antigen-negative wells was subtracted from each result, and the OD for each sample was corrected to a positive serum with the limit values of 2.0 OD in each run to generate a corrected optical density (COD) value. Serum samples from pre-immune mink and mice were used as a negative control with a COD value of <0.6 and <0.3, respectively.

### 2.5. Proliferation Assay

Spleens collected from the mice were processed for isolation of splenocytes three weeks after the second immunization. The freshly isolated splenocytes’ suspensions were counted and plated in 96-well cell culture plates at a density of 2 × 10^5^ cells/well in Roswell Park Memorial Institute (RPMI) and the medium supplemented with 5% of fetal calf serum, 0.2% of L-Glutamine, 100 Units/mL of penicillin, 100 µg/mL of streptomycin, and 0.1% of 2-mercaptoethanol. After overnight incubation at 37 °C, the cells were stimulated with 1, 2, 4, and 8 µg/mL of the corresponding MAstV CPs, in triplicate wells for each protein concentration. Cells from the sham mice were also stimulated in the same manner. Following incubation for 48 h at 37 °C, 5% CO_2_, the plates were centrifuged at 1200 rpm for 10 min, and the supernatants were removed and stored at −70 °C for determination of cytokines. For evaluation of proliferation, 96-well cell culture plates containing cell suspension were prepared and treated as before. Following incubation for 48 h at 37 °C, 5% CO_2_, the proliferation of specific cells was assessed using a water-soluble tetrazolium salt (WST-1) rapid Cell Proliferation kit (Calbiochem, San Diego, CA, USA). 10 µL of the WST-1 mixture, a colorimetric indicator of cell viability was added, and absorbance at 450 nm was measured with a spectrophotometer reader 2 h later.

### 2.6. Cytokine Profiling

The collected culture supernatants of the ex vivo-stimulated splenocytes were analyzed in triplicate using the mouse Th1/Th2 Cytokine Cytometric 6-plex Array Bead kit (Invitrogen, Carlsbad, CA, USA) that detects Interferon gamma (IFN-γ) and Interleukins (IL2, IL-4, IL-5, IL-10, and IL-12), according to the manufacturer’s protocol. Briefly, filter plates were pre-wetted, and 50 µL of coated bead suspension was added to each well and washed twice in a Tecan device. The samples and standards (50 µL) were then added in duplicate wells; the plates were sealed and shaken for 30 s at 1100 rpm and then incubated for 1 h, shaken at 300 rpm. The plates were washed three times, and 25 µL of diluted detection antibody was added to each well. The plates were shaken as before and then incubated for 30 min, shaken at 300 rpm in the dark. After washing three times, 50 µL of 1× streptavidin-Phycoerythrin (PE) was added to every well, and the plates were incubated for 10 min. The plates were washed again, and the beads were resuspended in 125 µL of the resuspension buffer, mixed by brief vertexing, and immediately read on the Luminex^®^ 100/200™ System (R&D Systems, Minneapolis, MN, USA) using xPONENT^®^ software (Luminex Corporation, Austin, TX, USA).

### 2.7. Statistical Analysis

The mean COD value and standard deviation (SD) of the T-cell proliferation test and the cell-mediated Luminex results of each group of immunized and sham mice were analyzed by using Student’s *t*-test (two-tailed distribution, two-sample unequal variance, Heteroscedastic) for differences among groups. Indeed, ELISA results were analyzed statistically using two-way analysis of variance (ANOVA) with replication test. The number of asterisks in figures show various significant differences between the variables: * for *p* < 0.05, ** for *p* < 0.01, and *** for *p* < 0.001. Comparison of secreted cytokine profiles measured by Luminex was performed using GraphPad Prism software version 4 (GraphPad Software, San Diego, CA, USA) and illustrated as heatmaps.

## 3. Results

### 3.1. Antibody Responses to MAstV CPs in Mice

**Figure 2:**
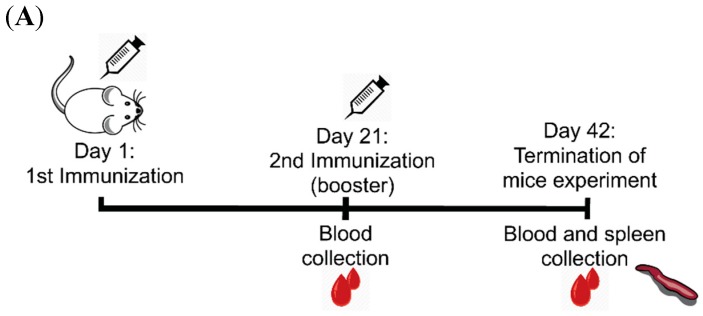

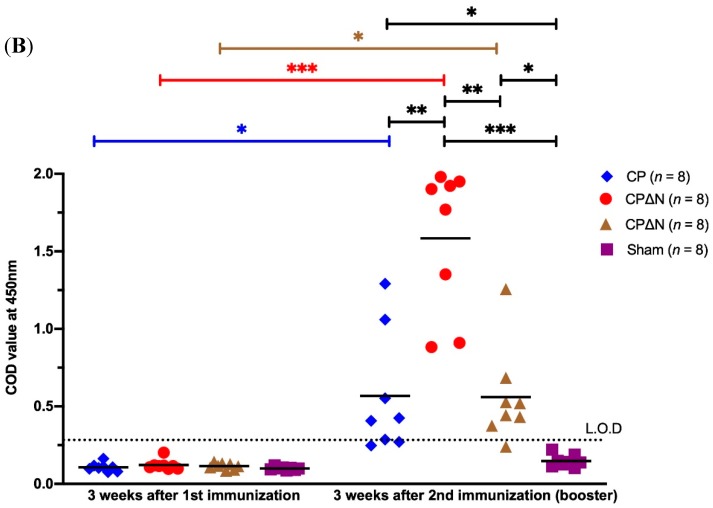
Schema of mice immunization study and indirect enzyme-linked immunosorbent assay (ELISA) titers of sera collected three weeks after the first and second immunization. (**A**) The MAstV vaccine candidates (5 µg/mouse) combined with an equal volume of AbISCO-100 adjuvant (10 µg/mouse) were injected subcutaneously to Naval Medical Research Institute (NMRI) mice (*n* = 8 per group, 4 weeks old) twice with a three-week interval. Mice injected with pDual-GC-vector-transfected cell lysate (5 µg/mouse) combined with adjuvant (10 µg/mouse) were analyzed as a sham group (*n* = 8). Spleen samples were collected at the end of the trial for ex vivo proliferation and cytokine analysis. (**B**) The serum samples of mice were collected three weeks after each immunization and tested with an indirect ELISA (Limit of detection (L.O.D) is the corrected optical density (COD) above 0.3). CP refers to the full-length capsid protein (spanning amino acids (aa) 1–775 of CP) of MAstV; CPΔN refers to an N-terminal truncated protein (spanning aa 161–775 of CP) of MAstV; CPΔC refers to a C-terminal truncated protein (spanning aa 1–621 of CP) of MAstV; Sham refers to the control group injected with pDual-GC-vector-transfected cell lysate. The mean value of each group (*n* = 8) of mice was calculated, the COD value of each mouse is illustrated in the dot plot. Asterisks indicate the level of significant difference between the mean value of either immunized or sham groups using two-way analysis of variance (ANOVA) with replication test (* *p* < 0.05, ** *p* < 0.01 and *** *p* < 0.001). Bars represent the mean value for each group.

Blood of mice immunized with CP, CPΔN, or CPΔC proteins and of sham mice (*n* = 8 per group) were collected three weeks after the first and second immunizations (Figure 2A). The sera were analyzed with an indirect ELISA for the detection of antibodies to MAstV CP. The antibody levels after the first immunization were low and without difference between the immunized mice groups and also between immunized and sham mice groups. After receiving the booster injection, the immunized mice showed a significant increase in anti-MAstV CP antibody levels (*p* < 0.05) compared to the first immunization. Interestingly, the antibody levels induced by CPΔN increased significantly (*p* < 0.001) after the booster and were markedly higher (*p* < 0.01) than antibodies generated by the booster of CP or CPΔC immunogens (Figure 2B). Although the antibody reactions to CP and CPΔC had similar mean COD values both after the first and booster immunizations, their mean COD values after the booster were significantly higher than sham (*p* < 0.05).

### 3.2. Proliferation Activity of Mice Splenocytes Immunized with MAstV CPs

**Figure 3:**
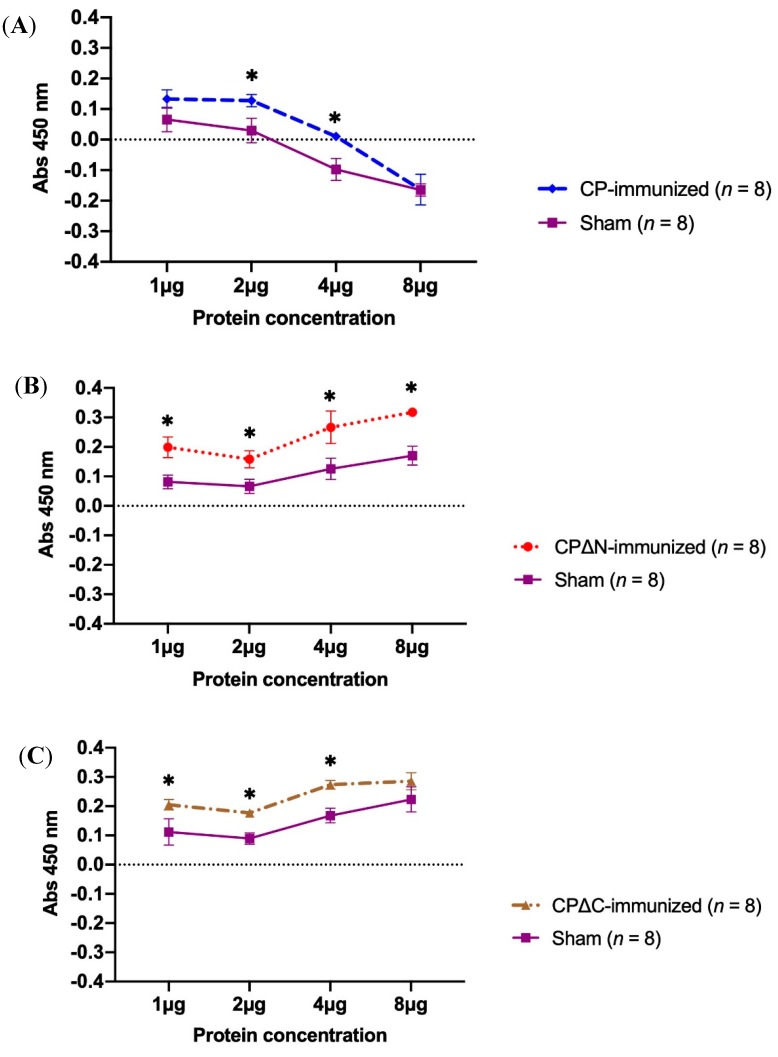
Proliferation assay of mice splenocytes after re-stimulation with different concentrations of the corresponding MAstV capsid proteins. The MAstV vaccine candidates (5 µg/mouse) combined with an equal volume of AbISCO-100 adjuvant (10 µg/mouse) were injected to NMRI mice (*n* = 8 per group, 4 weeks old) twice with a three-week interval. Mice injected with pDual-GC-vector-transfected cell lysate combined with adjuvant were also analyzed as sham (*n* = 8). Three weeks after the second immunization, the splenocytes were harvested from all mice groups: (**A**) CP-immunized (*n* = 8), (**B**) CP∆N-immunized (*n* = 8), and (**C**) CP∆C-immunized (*n* = 8). The splenocytes were then purified, cultivated ex vivo (2 × 10^5^ cells/well), and stimulated by exposing to different final concentrations (1 μg, 2 μg, 4 μg, and 8 μg) of the corresponding MAstV CPs as described in Materials and Methods; cell proliferation was measured in a WST-1 assay after 48 h of incubation. The splenocytes of the sham group (*n* = 8) were also independently exposed to each of the three MAstV CPs. The data are mean COD value readings of triplicate experiments. Asterisks indicate a significant difference at the given protein concentration between immunized and sham mice tested by two-way ANOVA with replication test (* *p* < 0.05). Error bars represent standard deviation (SD).

The viability of splenocytes using WST-1 labeling mixture was measured, showing different effects of various concentrations of MAstV CPs on splenocyte proliferation. Readings were indicative of lymphocyte proliferation recorded in supernatants from stimulated splenocytes in immunized but not in sham mice (Figure 3). Comparing to the sham group, the CP group did not induce splenocyte proliferation at higher than 4 µg/mL concentrations of MAstV CP (Figure 3). In contrast, CPΔN and CPΔC showed significant increases (*p* < 0.05) in proliferation ability in response to increased concentration of these MAstV CPs (Figure 3B,C). Splenocytes from sham mice, when stimulated with CPΔN or CPΔC, showed slightly higher proliferation at higher concentrations of these MAstV CPs (Figure 3B,C). However, increasing the CP concentration decreased the proliferation of splenocytes from sham mice too (Figure 3A).

### 3.3. Cytokine Profiling of MAstV CPs-Immunized Mice

**Figure 4:**
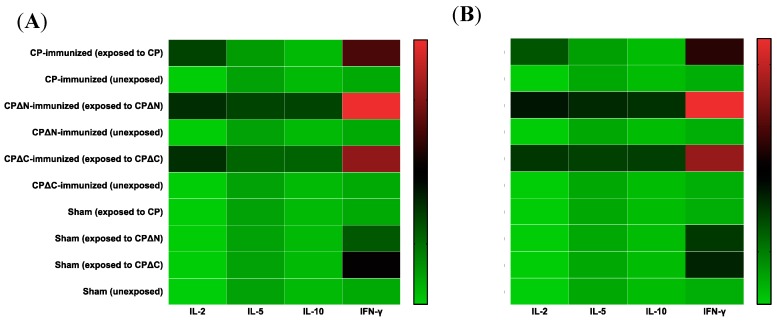
Heatmap of T-cell-mediated immune response to the corresponding MAstV CPs. The MAstV vaccine candidates (5 µg/mouse) combined with an equal volume of AbISCO-100 adjuvant (10 µg/mouse) were injected into NMRI mice (*n* = 8 per group, 4 weeks old) twice with a three-week interval. Mice injected with pDual-GC-vector-transfected cell lysate combined with adjuvant was also analyzed as a sham (*n* = 8). Three weeks after the second immunization, mice splenocytes were harvested from each group of mice: CP-immunized mice, CP∆N-immunized mice, and CP∆C-immunized mice. Mice injected with pDual-GC-vector-transfected cell lysate combined with adjuvant was also analyzed as a sham (*n* = 8). The splenocytes of four mice per group were then extracted, washed, and cultivated ex vivo (2 × 10^5^ cells/well) and stimulated by exposing to (**A**) 2 µg/mL and (**B**) 4 µg/mL of the corresponding MAstV CPs as described in Materials and Methods. The culture supernatants from stimulated splenocytes were collected and assessed for secreted cytokines IL-2, IL-5, IL-10, and IFN-γ by using a mouse Th1/Th2 Cytokine Cytometric 6-plex Array Bead kit (Invitrogen), Luminex^®^ 100/200™ System and xPONENT^®^ software (Luminex Corporation, Austin, TX, USA). The splenocytes of the sham group (*n* = 8) were also independently exposed to each of the three MAstV CPs. The data are mean COD readings of duplicate experiments, illustrated as log 10 values in the heatmap.

Next, we investigated the capacity of different generated MAstV CPs to induce specific Th1 and Th2 responses. The culture supernatants from stimulated splenocytes with 2 and 4 µg/mL concentrations of MAstV CPs were assessed for secreted cytokines using the mouse Th1/Th2 Cytokine Cytometric 6-plex Array Bead kit (Invitrogen, Carlsbad, CA, USA) that measures IFN-γ, IL-2, IL-4, IL-5, IL-10, and IL-12. The results are displayed as heatmaps (Figure 4A,B). For IL-2, a significant readout was found in mice immunized with all three proteins. Indeed, the readouts in mice immunized with truncated CPs were found higher than full-length CP and were the highest in the CPΔN-immunized mice group when exposed to a higher (4 µg/mL) concentration of this immunogen (Supplementary Figure S1A). For IL-4, the measured values were, in general, low with a minor difference between CP and the two CP truncates (data not shown). IL-5 and IL-10 responses showed a similar pattern to IL-2, with higher and significant stimulation induced in mice immunized with CPΔN (Supplementary Figure S1B,C). Furthermore, mice immunized with CP did not show any difference between IL-5 and IL-10 responses (Supplementary Figure S1B,C). For IL-12, there were no differences between the readings of immunized and sham mice showing that there is no induction of this cytokine by any of MAstV CPs (data not shown). No significant changes in cytokine levels were observed in sham mice for the investigated interleukins (Figure 4). For IFN-γ, significantly super high levels of cytokine were induced in immunized mice when stimulated with CPΔN (*p* < 0.001); four folds at 2 µg/mL and eight folds at 4 µg/mL concentration of CPΔN (Supplementary Figure S1D). Also, readings of IFN-γ from CPΔC (*p* < 0.05), though not as high as CPΔN were higher than those from CP at increasing concentrations of this immunogen: One-fold at 2 µg/mL and three folds at 4 µg/mL concentration of CPΔC (Supplementary Figure S1D). Interestingly, naïve mice in the sham group when stimulated with the different concentrations of only CPΔN and CPΔC also showed a higher level of expression for IFN-γ response (Figure 4), showing the capability of the two AstV CP truncates in immunogenicity in naïve mice.

Though inducing a low level of antibodies, the CPΔC is endowed with better capacity for mounting a recall response by means of strong cellular immunity, compared to the full-length CP. The CPΔN protein of AstV combines both the ability to induce high levels of antibodies (humoral response) with the capacity to stimulate cytokine production (cellular response) thereby stimulating both arms of the immune response. In general, the production of cytokines increased with the amount of MAstV CPs used in the re-stimulation of splenocytes. Altogether, the results showed a poor ability of CP to stimulate cellular responses based on the measured cytokines. On the other hand, the findings also reveal differences in the degree of cytokine induction between CPΔN and CPΔC, albeit the fact that they both stimulate cellular responses. The practical significance of this circumstance in full protection was also evaluated in challenge experiments in mink litters.

**Figure 5:**
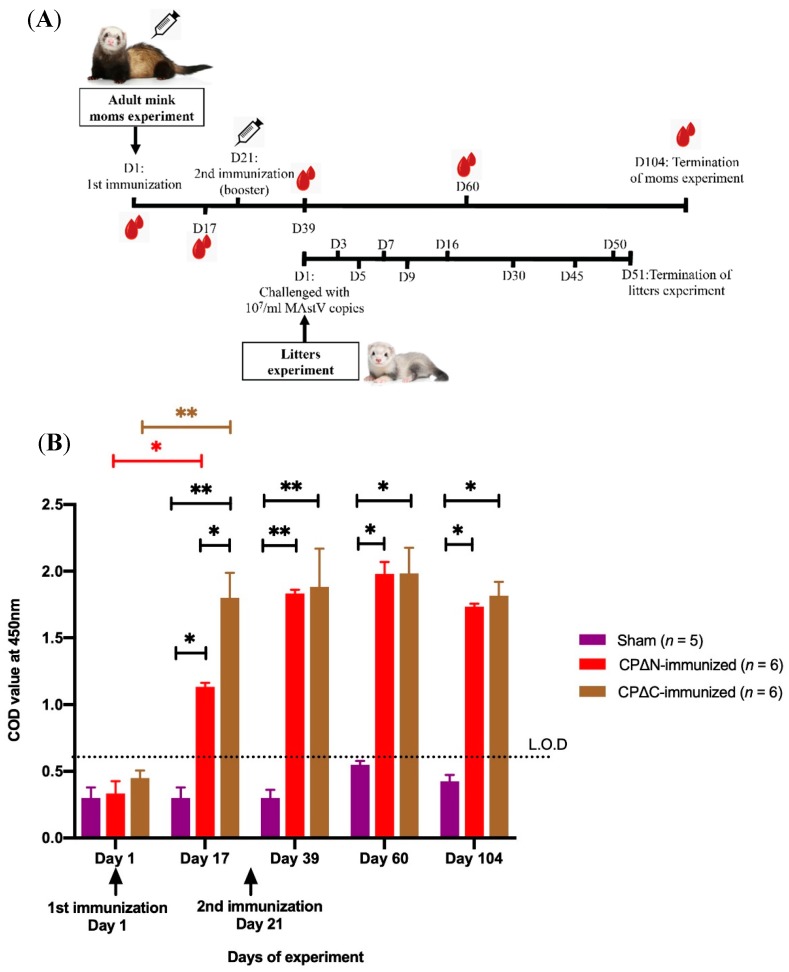
Schema of mink immunization and ELISA of the humoral immune response to mink astrovirus capsid protein (MAstV CP) vaccine candidates in adult mink moms. (**A**) The MAstV vaccine candidates (100 µg/animal) combined with an equal amount of Freund’s adjuvant were injected into female adult minks (*n* = 6 per group) twice with three weeks interval. The adult moms injected with pDual-GC-vector-transfected cell lysates combined with adjuvant were also analyzed as a sham (*n* = 5). On day (D) 1 after birth, the litters (*n* = 89) of these immunized and sham adult mink moms (*n* = 17) were then challenged orally with 10^7^/mL MAstV copies. As a follow-up, the fecal samples of their newborn litters on test days 1, 3, 5, 7, 9, 16, 30, 45, 50, and 51 after birth were tested using real-time PCR for detecting astrovirus shedding status. The clinical signs of litters were also monitored and recorded by veterinarians every other day. (**B**) Serum samples of the adult moms were collected on test days 1, 17, 39, 60, and 104 and tested with an indirect ELISA for measuring maternal antibody production against MAstV (Limit of detection (L.O.D) is the corrected optical density (COD) above 0.6). CPΔN refers to an N-terminal truncated protein (spanning aa 161–775 of CP) of MAstV; CPΔC refers to a C-terminal truncated protein (spanning aa 1–621 of CP) of MAstV; Sham refers to the control group injected with pDual-GC-vector-transfected cell lysate. Asterisks indicate significant differences at the given protein concentration between various immunized and sham adult mink moms tested by a two-way ANOVA with replication test (* *p* < 0.01, ** *p* < 0.001). Error bars represent SD.

Maternal passive immunization with truncated MAstV CPs was done in mink. A total of 85 serum samples were taken from MAstV CPs-immunized female adult minks (*n* = 17) on five occasions during a 104-day experiment (Figure 5A). Using ELISA, the levels of anti-MAstV CP antibody in serum samples were measured. The ELISA results of each MAstV vaccine candidate are shown in Figure 5B. Both CPΔN- and CPΔC-vaccinated groups generated significantly higher levels of antibodies after the first and second immunization, compared to the sham group (*p* < 0.01), with a highest (*p* < 0.001) anti-MAstV CP antibody titer three weeks after the second immunization (day 39; Figure 5B). Furthermore, though a slight decrease, these vaccinated adult females showed a significantly higher level of anti-MAstV CP antibody (*p* < 0.01) than the naïve sham group (Figure 5B) during the entire trial. The antibody levels, generated after the first immunization with CPΔC-vaccinated candidates, were significantly higher than the CPΔN-vaccinated group (*p* < 0.01) as measured on day 17 (Figure 5B). However, this level after the second immunization with CPΔN was measured slightly higher than that of the CPΔC-vaccine candidate (Figure 5B) as measured on day 39.

**Figure 6:**
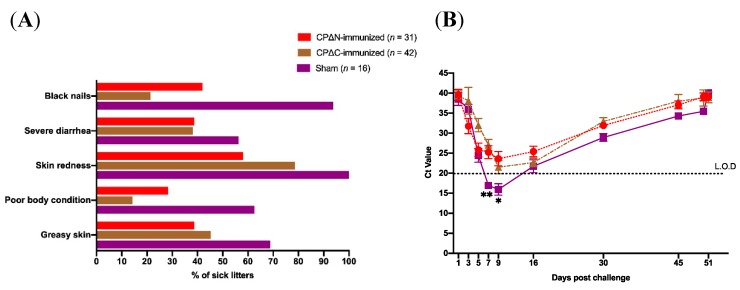
Graphs of the results of the real-time polymerase chain reaction (PCR) and clinical signs observed in the experiment of litter minks. After birth, the one-day newborn litters (*n* = 89) of immunized and sham adult moms (*n* = 17) were challenged with 10^7^/mL MAstV copies. As a follow-up, the fecal samples (*n* = 414) of their newborn litters on test days 1, 3, 5, 7, 9, 16, 30, 45, 50, and 51 post-challenge were tested using a real-time PCR for detecting AstV shedding status. The clinical signs of litters were also monitored and recorded by the author veterinarians every other day. (**A**) The ratios of litters showing various clinical signs to total group-related litters were calculated and illustrated as a percentage (%) in the graph. (**B**) The mean cycle threshold (Ct) value of real-time PCR results of sampled litters on each sampling occasion during challenge experiment (51 days) was calculated and is illustrated in the graph. Asterisks show a significant decline of virus shedding among litters of either both CP∆N- and CP∆C- (**) or just CP∆N- (*) immunized adult mom groups compared to those of the sham group using the Log-rank test (*p* < 0.05). Bars indicate the standard deviation (SD).

After the challenge of the mink litters (on day 1 with 10^7^ MAstV genome copies of a heterologous strain), the clinical signs and virus shedding of their litters were investigated. The litters showed clinical signs including poor body condition, skin redness, severe diarrhea, and black nails, although the vaccinated groups showed milder severity of clinical signs compared to the sham group (Figure 6A). Furthermore, the ratio of litters with either clinical signs of AstV infection declined among vaccinated groups compared to the sham group (Figure 6A). Only 14.3% of litters (*n* = 42) in CPΔC and less than half (48.4%) of litters (*n* = 31) in CPΔN groups showed poor body conditions. While more than half (56.2%) of litters (*n* = 16) in the sham group showed severe diarrhea on days 7–14 post-challenge, only around 38% of litters (12 out of 31) in CPΔN and (16 out of 42) in CPΔC groups showed mild to severe diarrhea (Table 2). Greasy skin signs were observed in 68.7% (11 out of 16) of litters in the sham group, while this main clinical sign of MAstV infection was observed only in 38.7% (12 out of 31) of litters in CPΔN group and 45.2% (19 out of 42) of litters in CPΔC group. For black nail sign rates of MAstV infection, the litters in CPΔN and CPΔC groups showed around 50% and 70% lower rates, respectively, compared to those in the sham group (Table 2).

Totally, 414 fecal samples of all 89 litters (87 samples from the sham moms’ litters (*n* = 16), 146 from CPΔN-vaccinated moms’ litters (*n* = 31), and 181 from CPΔC-vaccinated moms’ litters (*n* = 42)) were collected on nine occasions and tested by PCR for shedding MAstV detection during the 51-day challenging experiment in litters (Table 1). On the day of challenge (day 1) and day 3, all litters were tested to find the accurate time that MAstV shedding starts. To follow up all litters during this experiment, two litters of each adult mom were tested using a real-time PCR on each sampling occasion. The ratio of positive samples to the total number of tested litters of each group on a sampling occasion was calculated and presented as a percentage. The results showed that on day 7 post-challenge, the rate of virus-shedding among the litters in both CPΔN- and CPΔC-vaccinated mom groups significantly declined (>40%) compared to that of the sham mom group (*p* < 0.05) (Figure 6B, Supplementary Figure S2). This significantly lower rate (40%) of virus-shedding prolonged to day 9 just in the CPΔN-vaccinated moms’ litters, and stayed lower than those in CPΔC-vaccinated moms (by 30%) and the sham moms (by 40%) groups. In this study, at the peak of virus shedding (day 16), when all (100%) litters in the CPΔC-vaccinated moms group (*n* = 12), and sham moms groups (*n* = 8) were detected positive, almost 20% of litters (*n* = 11) in the CPΔN-vaccinated moms group were found free from MAstV infection. Furthermore, the remaining sampled litters (6 out of 9) in the CPΔN group and most of the sampled litters (9 out of 12) in CPΔC group on day 16 were detected with a lower rate (Ct value > 30, corresponding to 10^3^ RNA copies of MAstV) of MAstV shedding using a quantitative PCR [36] compared to sham group in which all litters (8 out of 8) were detected with a high rate (Ct value < 20, corresponding to 10^6^ RNA copies of MAstV) of virus shedding in their fecal samples. Also, the period of virus shedding in litters was recorded between 3–50 days post-challenge (Figure 6B, Supplementary Figure S2). Collectively, the litters in both CPΔN- and CPΔC-vaccinated moms groups showed milder rates of all investigated clinical signs and also lower rates of virus shedding post-challenge, with a high dose of heterogeneous MAstV strain DK7627 (10^7^ virus genome copies), when compared with the litters in the sham moms group. This is also in correlation with a higher rate of MAstV seropositivity of their mink moms immunized with CP truncates, compared to full-length CP and the sham group (Table 3). This indicates the protective effect of maternal immunization with truncated-MAstV CPs vaccine candidates. Indeed, anti-MAstV antibodies generated by this strategy could pass to litters during and/or after gestation and could neutralize MAstV post-challenge.

## 4. Discussion

Infection with gut viruses such as rotaviruses, AstV, and noroviruses (Norwalk virus, calicivirus) are spread worldwide in human, animal, and bird species with a high seroprevalence [38,39,40,41]. Despite remarkably growing studies on AstV epidemiology and molecular biology [41,42], little is known about its immunogenicity and vaccine development. Recent findings about AstVs from different species highlighted high seroprevalence [8,25,38,39], neurotropism [29,30], zoonotic transmission [26], and huge economic losses [8,31,32,39]. These raise the importance of an efficient vaccine for this infection, not only for the animal industry but also for public health concerns. Currently, there are no vaccines or antiviral therapies against AstV diseases in either public health or animal health, which persuaded us to research and to provide knowledge on vaccine design and immunization strategies against AstVs. In several phylogenetic analysis studies, MAstV strains have been clustered to its human counterpart [38,43]. Recent advanced sequence analyses have also identified two novel groups of highly divergent AstVs, named MLB (Melbourne) and VA/HMO (Virginia/Human-Mink-Ovine-like) in human individuals with diarrhea [44,45]. Furthermore, in clinical studies including the authors’ observations, there is evidence of MAstV-associated encephalitis and meningitis in minks and humans raising the zoonotic emerging aspect of MAstV [26]. These studies highlight the importance of MAstV vaccine research.

In this study, significantly higher anti-MAstV serum antibody detected in mice and mink showed that CP truncates could elicit a reasonable systematic humoral immune response compared to full-length CP. Humoral immunity plays an important role in protecting against AstVs since the presence of protective antibodies against HAstV in healthy adults provides a mechanism of protection against reinfection [46,47]. The development of anti-AstV therapies is hampered by the gap in knowledge of protective antibody epitopes on the AstV capsid surface [48] and the immunosuppressing peptides along AstV CP [46,49,50]. Most of AstV vaccine research studies, including our previous study, have been focused on the full-length CP vaccine candidates, which all showed partial unsatisfactory immunity [36,37,51]. Our previous study, however, supports the feasibility of providing a truncated AstV CP vaccine for protection against AstV infection [36]. The most recent trial was also relevant to characterize a humoral immune response to a trivalent vaccine containing VP26 of the HAstV CP immunization in mice [52].

Currently, there is no commercial immunological kit available to evaluate the cytokine levels in mink to get a better overview of immune responses following administration of mink vaccine candidates. Thus, we administered these MAstV vaccine candidates subcutaneously in adult NMRI immunocompetent mice to evaluate both humoral and cellular Th1/Th2 cellular immune responses. The MAstV CP truncates evaluated in this study could drastically induce higher IFN-γ secretion in mice splenocytes compared to the full-length CP (Figure 4, Supplementary Figure S1D). This is indicative of the role of full-length CP in depleting IFN-γ, type II interferon, a mechanism which is exploited by AstV to promote viral replication in early infection. AstVs are IFN-sensitive viruses that so far have been shown to induce IFN-β, type I interferon, which occurs late in infection and is independent of replication [53]. This limits AstV infection and preserves barrier permeability in the intestines [53,54]. Some other gut viruses such as rotavirus exploit IFN signaling in intestinal cells to promote early viral replication [55,56]. Later on, IFN-induced apoptosis helps to control these viral infections. Our findings show that truncation disrupts this antiviral suppression activity of AstV CP, leading to better crosstalk between humoral and cellular immune responses.

Turkey AstV infection is a poor inducer of an adaptive immune response [57] and resulted in reinfection. Studies using the newly emerging mouse model and clinical studies in humans also demonstrated that the adaptive immune response is key in controlling AstV infection [58], which has been shown not to be well induced by whole AstV or full-length CP [36,48]. A study by Bogdanoff, et al. has revealed that the CP core domain (VP34) is antigenic in addition to the CP spike domains (VP27/29 and VP25/26) [47]. CP∆N truncate (aa 161–775) in this study, despite of lacking the N-terminus (aa 1–80) and partial inner core domain (aa 80–266) of CP, showed higher adaptive humoral and cellular immune responses compared to CP and CP∆C. Previously, immune-suppressing mechanisms of a peptide (aa 79–139) along N-terminus of AstV through interacting with a complement system has been shown [49,50]. This supportive reason indicates that CP∆N also could not interrupt the crosstalk between innate and adaptive immune responses due to the lack of suppression capability of the complement system; therefore, it is the most immunogenic vaccine candidate in this study. Such immunosuppressing mechanism ruled out by N-terminus peptide of CP∆C truncate (aa 1–621) may likely be a reason for the lower cytokine induction in mice splenocytes, indicating a lesser capability of this truncate to elicit adaptive immune responses after immunization, compared to the CP∆N truncate.

Notably, CP∆C also showed a higher cellular immune response after re-stimulating mice splenocytes and higher humoral immune response in the mink model compared with full-length CP. These results support a hypothesis that AstV CP has more mechanisms for immunosuppression than the one for suppressing the complement system, which requires more investigation. Genomic [59] and proteomic [49,60] studies have shown that conserved non-immunogenic regions are located at N- and C-termini of CP. The innate immune system is the first line of defense against an invading pathogen. Some mediators of innate immune systems such as active transforming growth factor beta (TGF-β) levels and synthesis of inducible nitric oxide synthase (iNOS) protein are increased after AstV infection, but the role of TGF-β after AstV infection is not known [38,61]. The presence of signal transducer and activator of transcription 1 (STAT1) also decreases AstV replication with its mechanism remaining unknown. Due to these innate immunosuppressing mechanisms of AstV, its infection is associated with mild levels of histopathological signs, destruction of intestinal villi, and non-inflammatory diarrhea in calves, lambs, and turkey case studies [62,63,64,65]. Our study also shows that AstV CP truncates could stimulate some levels of secretion of IL-10 that is related to TGF-ß signaling. However, we were unable to measure secreted TGF-β in mice splenocytes after stimulation with full-length and the truncated AstV CP immunogens. Taken together, research on finding extra immunosuppressing peptides along AstV CP truncates and removing them from current CP truncates would indispensably benefit AstV vaccinology by providing better crosstalk between innate and adaptive immune responses to elicit even better adaptive immunity after vaccination.

Due to difficulties of litter vaccination, our ultimate goal of an MAstV vaccine is to provide maternal passive immunity in litters by vaccinating adult female moms. Ideally, maternal passive immunity observed among litters of mink refers to anti-AstV CP IgG that can pass to litters through the placenta during gestation and/or through the milk after birth. The induction of humoral immunity characterized by the stimulation of B-lymphocytes in moms is then critical in this work to generate a protective level of anti-AstV IgG antibodies in newborn litters. The passive protection of mink litters born to immunized moms after challenge with AstV -positive fecal filtrate was evaluated by the investigation of clinical signs and virus shedding. Although the litters were challenged with a high dose (10^7^ genome copies) of a heterogeneous strain DK7627 which is known to be a causative agent of shaking mink syndrome [28], the results indicated the presence of protective maternal anti-AstV IgG antibodies in litters. Maternal passive immunization evaluated in the mink trial of this study supports the application of such an immunization with a safe (gene- or protein-based) subunit AstV vaccine to protect newborns after gestation. Maternal passive immunization has been used for vaccination against other infections such as influenza virus [66,67], enterovirus [68,69], respiratory syncytial virus [70,71], pertussis [72,73], group B streptococcus [74], tetanus [75,76], thereby showing that immunization during pregnancy is the most effective means of preventing maternal and newborns mortality/morbidity. Safety of vaccination in pregnancy, however, is a key consideration. Gene- and protein-based subunit vaccines are classified among the safest types of vaccines and have always been considered for maternal vaccination. These are effective strategies to prevent serious infections in mothers and their infants in both veterinary and human medicine.

Truncated forms of MAstV CP could abate the virus shedding rate and clinical signs of AstV infection in litters of immunized groups compared to the sham group. However, the AstV infection could not be prevented completely by vaccination. The overdosing of virus inoculation of a heterologous MAstV strain could be a reason for still observing virus shedding and diarrhea in some litters of the vaccinated groups. Isolation and titration of AstVs have been difficult due to viral characteristics [77]. In the same way, our attempts to prepare a titrated infectious solution to be used for MAstV inoculation failed. Lack of such a convenient infectious solution could overdose virus inoculation in mink litters, leading to underestimating the protective efficacy of vaccines. Reduction in clinical signs showed that maternal immunization produced effective neutralizing antibodies to protect litters against MAstV infection.

The capacity to elicit an effective T- and B-lymphocyte immunity can be shown by the stimulation of lymphocyte proliferation response, antibody titration, and cytokine profiling. In this work, we show that truncated forms of AstV CP were the stronger inducers of antibody secretion and lymphoproliferation. Increase of IL-2 and WST-1 tests showed a stronger ability of CP truncates to stimulate the proliferation and differentiation of T cells, which is important for local and systematic humoral immunity [46]. Although no increase of cytokines was detected in splenocytes of the sham mice group, CPΔC and CPΔN could elicit Th1 (IL-2 and IFN-γ), Th2 (IL-5), and Th2/T-reg (IL-10) in all immunized mice, with the highest level of induction in the CPΔN-immunized group. It is well known that Th1-associated cytokines help to regulate antiviral cellular responses, while Th2-associated cytokines enhance humoral immune responses [38,46]. Subsequently, the level of serum neutralizing polyclonal antibodies also significantly increased after the second immunization with both CP truncates. We showed that CP truncates could better elicit adaptive humoral and cellular responses compared to full-length CP. In contrast, immunization with MAstV CP raised a weak level of cytokines and antibodies in mice, which is in accordance with previous field studies [36,37,51]. It is now well documented that cooperation between cellular and humoral immunity is indispensable to provide a protective immune response against numerous pathogens [78], including AstVs [46,62]. Taken together, combined with analyses of serum antibody levels, these data suggest that a better balanced Th1/Th2 immune response is induced by the truncates of MAstV CP, compared to the suboptimal protection by full-length CP.

In this study, we did not collect intestine samples to measure the mucosal immune response that may conceivably mediate protection against AstV infections. A previous study showed that CD4+ T cells that reside in the duodenal mucosa are presumably important in mucosal defense against recurrent AstV infections [79]. Vaccine trials of CP of norovirus and rotaviruses in mice with similar Th1/Th2 immune responses could elicit mucosal immunity [51,80,81]. Since local mucosal immunity is believed to play an important role in protection against viral infections in the gastrointestinal tract, an ideal vaccine would induce strong mucosal immunity [82]. Thus, further studies are required to find out whether mucosal immune responses in the intestines could be elicited by vaccine administration with CP truncates.

Due to the unavailability of MAstV challenge in mice, it remains unknown whether the immunity evoked by subcutaneous MAstV CP immunization is capable of protecting against AstV infection. However, we may anticipate that it would have a protective effect based on knowledge obtained from studies of other similar viral infections like rotavirus and norovirus [51,80,81]. To date, young turkeys (poults) infected with turkey AstV and mice (C57BL/6, IFNαR^−/−^) infected with murine AstV are the best-defined animal models for AstV pathogenesis [54,62]. A 2016 study showed that wild type mice infected with a fecal filtrate containing 2 × 10^7^ genomic copies of murine AstV began shedding virus within 2 days post-challenge, and viral titers reached a peak of 10^7^ copies/µg RNA between 6 and 11 days post-challenge before decreasing over the remainder of the study, ultimately clearing by 53 days post-challenge [54]. Interestingly, this pattern of AstV shedding is so similar to our results for mink litters (*n* = 16) of the sham group (Figure 6B). Almost no study has been reported on MAstV pathogenesis and vaccine development. High genetic identity and clinical sign similarities between mink and human AstVs could allow mink to be a worthy animal model for studying AstV immunology and vaccine development. The results of this present study could provide us a new approach for human AstV vaccine development.

## 5. Conclusions

We characterized humoral and cellular immune responses in a mouse model immunized with not only AstV CP full-length but also AstV CP truncates as novel subunit vaccine candidates for AstV infection. This study shows that lymphocytes T-helper cells provided help for effective humoral immunity. Furthermore, we showed that optimal protection was given from maternal passive neutralizing antibodies induced by two novel AstV subunit vaccines based on an N- and C-terminally truncation strategy along AstV CP in a mink model. Further studies are required to distinguish the immunosuppressing motifs from immunogenic epitopes and remove them from vaccine candidates, thereby, leading to better adaptive immune responses. These conclusively support this idea that the truncation strategy along AstV CP and passive immunization regime described in this study could also be used to develop safe and effective subunit (proteomic or genomic) vaccine candidates not only for animal health but also for human infants, elderlies, and immunocompromised populations.

## Figures and Tables

**Table 1:** Summarizing the data of various groups in the mink trial.

Group of Experiment	No. of Adult Female Minks (%)	No. of Litters (%)	No. of Samples Tested by PCR (%)
Sham ^1^	5 (30)	16 (18)	87 (21)
CPΔN ^2^ -immunized	6 (35)	31 (35)	146 (35)
CPΔC ^3^ -immunized	6 (35)	42 (47)	181 (44)
Total	17 (100)	89 (100)	414 (100)

Note: ^1^ Sham refers to the control group injected with pDual-GC-vector-transfected cell lysate; ^2^ CPΔN refers to an N-terminal truncated protein (spanning aa 161–775 of CP) of MAstV; ^3^ CPΔC refers to a C-terminal truncated protein (spanning aa 1–621 of CP) of MAstV.

**Table 2:** Summarizing the evaluated clinical sign observation of litters in mink trial.

Group of Experiment	No. of Litters (%)	Evaluated Clinical Signs in Litters				
		Greasy Puppies (%)	Poor Body Condition (%)	Skin Redness (%)	Severe Diarrhea (%)	Black Nails (%)
Sham ^1^	16 (18)	11 (68.7)	10 (62.5)	16 (100)	9 (56.2)	15 (90.4)
CPΔN ^2^ -immunized	31 (35)	12 (38.7)	15 (48.4)	18 (58.1)	12 (38.7)	13 (42)
CPΔC ^3^ -immunized	42 (47)	19 (45.2)	6 (14.3)	33 (78.6)	16 (38.1)	9 (21.4)

Note: ^1^ Sham refers to the control group injected with pDual-GC-vector-transfected cell lysate; ^2^ CPΔN refers to an N-terminal truncated protein (spanning aa 161–775 of CP) of MAstV; ^3^ CPΔC refers to a C-terminal truncated protein (spanning aa 1–621 of CP) of MAstV.

**Table 3:** Seropositivity ratio of animal models 3 weeks after the first and second immunization during in vivo trials.

Group of Experiment	Ratio of Seropositivity (%)			
	3 Weeks after First Immunization		3 Weeks after Second Immunization (Booster)	
	Mice	Mink	Mice	Mink
Sham ^1^	0/8 (0)	0/5 (0)	0/8 (0)	0/5 (0)
CP ^2^ -immunized	0/8 (0)	NT ^3^	5/8 (62.5)	NT
CPΔN ^4^ -immunized	0/8 (0)	5/6 (83.3)	8/8 (100)	6/6 (100)
CPΔC ^5^ -immunized	0/8 (0)	5/6 (83.3)	7/8 (87.5)	6/6 (100)

Note: ^1^ Sham refers to the control group injected with pDual-GC-vector-transfected cell lysate; ^2^ CP refers to the full-length capsid protein (spanning amino acids (aa) 1–775 of CP) of MAstV; ^3^ NT—Not tested; ^4^ CPΔN refers to an N-terminal truncated protein (spanning aa 161–775 of CP) of MAstV; ^5^ CPΔC refers to a C-terminal truncated protein (spanning aa 1–621 of CP) of MAstV.

## Supplementary Materials

The following are available online at https://www.mdpi.com/2076-393X/7/3/79/s1, Figure S1: Analyses of T cell-mediated immune response to the corresponding MAstV CPs., Figure S2: Graph of results of real-time PCR and clinical sign observation in experiment of litters mink trial.

## References

[1] Holmgren J., Svennerholm A.M. (2012). Vaccines against mucosal infections. Curr. Opin. Immunol..

[2] Plotkin S.A. (2003). Vaccines, vaccination, and vaccinology. J. Infect. Dis..

[3] Suschak J.J., Williams J.A., Schmaljohn C.S. (2017). Advancements in DNA vaccine vectors, non-mechanical delivery methods, and molecular adjuvants to increase immunogenicity. Hum. Vaccines Immunother..

[4] Tan M., Jiang X. (2017). Recent advancements in combination subunit vaccine development. Hum. Vaccines Immunother..

[5] Humphreys I.R., Sebastian S. (2018). Novel viral vectors in infectious diseases. Immunology.

[6] Yang Y.T., Chow Y.H., Hsiao K.N., Hu K.C., Chiang J.R., Wu S.C., Chong P., Liu C.C. (2016). Development of a full-length cDNA-derived enterovirus A71 vaccine candidate using reverse genetics technology. Antivir. Res..

[7] Pardi N., Hogan M.J., Porter F.W., Weissman D. (2018). mRNA vaccines—A new era in vaccinology. Nat. Rev. Drug Discov..

[8] Englund L., Chriel M., Dietz H.H., Hedlund K.O. (2002). Astrovirus epidemiologically linked to pre-weaning diarrhoea in mink. Vet. Microbiol..

[9] Geigenmuller U., Chew T., Ginzton N., Matsui S.M. (2002). Processing of nonstructural protein 1a of human astrovirus. J. Virol..

[10] Mendez E., Salas-Ocampo M.P., Munguia M.E., Arias C.F. (2003). Protein products of the open reading frames encoding nonstructural proteins of human astrovirus serotype 8. J. Virol..

[11] Mittelholzer C., Hedlund K.O., Englund L., Dietz H.H., Svensson L. (2003). Molecular characterization of a novel astrovirus associated with disease in mink. J. Gen. Virol..

[12] Lewis T.L., Greenberg H.B., Herrmann J.E., Smith L.S., Matsui S.M. (1994). Analysis of astrovirus serotype 1 RNA, identification of the viral RNA-dependent RNA polymerase motif, and expression of a viral structural protein. J. Virol..

[13] Monroe S.S., Jiang B., Stine S.E., Koopmans M., Glass R.I. (1993). Subgenomic RNA sequence of human astrovirus supports classification of Astroviridae as a new family of RNA viruses. J. Virol..

[14] Willcocks M.M., Brown T.D., Madeley C.R., Carter M.J. (1994). The complete sequence of a human astrovirus. J. Gen. Virol..

[15] Mendez E., Aguirre-Crespo G., Zavala G., Arias C.F. (2007). Association of the astrovirus structural protein VP90 with membranes plays a role in virus morphogenesis. J. Virol..

[16] Toh Y., Harper J., Dryden K.A., Yeager M., Arias C.F., Mendez E., Tao Y.J. (2016). Crystal Structure of the Human Astrovirus Capsid Protein. J. Virol..

[17] Donelli G., Superti F., Tinari A., Marziano M.L. (1992). Mechanism of astrovirus entry into Graham 293 cells. J. Med. Virol..

[18] Bridger J.C., Hall G.A., Brown J.F. (1984). Characterization of a calici-like virus (Newbury agent) found in association with astrovirus in bovine diarrhea. Infect. Immun..

[19] Jonassen C.M., Jonassen T.T., Sveen T.M., Grinde B. (2003). Complete genomic sequences of astroviruses from sheep and turkey: Comparison with related viruses. Virus Res..

[20] Indik S., Valicek L., Smid B., Dvorakova H., Rodak L. (2006). Isolation and partial characterization of a novel porcine astrovirus. Vet. Microbiol..

[21] Hoshino Y., Zimmer J.F., Moise N.S., Scott F.W. (1981). Detection of astroviruses in feces of a cat with diarrhea. Brief report. Arch. Virol..

[22] Toffan A., Jonassen C.M., De Battisti C., Schiavon E., Kofstad T., Capua I., Cattoli G. (2009). Genetic characterization of a new astrovirus detected in dogs suffering from diarrhoea. Vet. Microbiol..

[23] Rivera R., Nollens H.H., Venn-Watson S., Gulland F.M., Wellehan J.F. (2010). Characterization of phylogenetically diverse astroviruses of marine mammals. J. Gen. Virol..

[24] Pantin-Jackwood M.J., Strother K.O., Mundt E., Zsak L., Day J.M., Spackman E. (2011). Molecular characterization of avian astroviruses. Arch. Virol..

[25] Koci M.D., Schultz-Cherry S. (2002). Avian astroviruses. Avian Pathol..

[26] Quan P.L., Wagner T.A., Briese T., Torgerson T.R., Hornig M., Tashmukhamedova A., Firth C., Palacios G., Baisre-De-Leon A., Paddock C.D. (2010). Astrovirus encephalitis in boy with X-linked agammaglobulinemia. Emerg. Infect. Dis..

[27] Vu D.L., Bosch A., Pinto R.M., Guix S. (2017). Epidemiology of Classic and Novel Human Astrovirus: Gastroenteritis and Beyond. Viruses.

[28] Baule C., Bidokhti M.R., Chriel M., Czifra G., Dietz H.H., Hammer A.S., Sandbol P., Ullman K. (2012). Recombinant Proteins as Vaccines for Protection against Disease Induced by Infection with Mink Astrovirus. Patent.

[29] Pfaff F., Schlottau K., Scholes S., Courtenay A., Hoffmann B., Hoper D., Beer M. (2017). A novel astrovirus associated with encephalitis and ganglionitis in domestic sheep. Transbound. Emerg. Dis..

[30] Bouzalas I.G., Wuthrich D., Walland J., Drogemuller C., Zurbriggen A., Vandevelde M., Oevermann A., Bruggmann R., Seuberlich T. (2014). Neurotropic astrovirus in cattle with nonsuppurative encephalitis in Europe. J. Clin. Microbiol..

[31] Schneider R.R., Hunter D.B. (1993). Mortality in mink kits from birth to weaning. Can. Vet. J..

[32] Gavier-Widen D., Brojer C., Dietz H.H., Englund L., Hammer A.S., Hedlund K.O., Hard af Segerstad C., Nilsson K., Nowotny N., Puurula V. (2004). Investigations into shaking mink syndrome: An encephalomyelitis of unknown cause in farmed mink (*Mustela vison*) kits in Scandinavia. J. Vet. Diagn. Investig..

[33] Perot P., Lecuit M., Eloit M. (2017). Astrovirus Diagnostics. Viruses.

[34] Bass D.M., Qiu S. (2000). Proteolytic processing of the astrovirus capsid. J. Virol..

[35] Dong J., Dong L., Mendez E., Tao Y. (2011). Crystal structure of the human astrovirus capsid spike. Proc. Natl. Acad. Sci. USA.

[36] Bidokhti M.R., Ullman K., Jensen T.H., Chriel M., Mottahedin A., Munir M., Andersson A.M., Detournay O., Hammer A.S., Baule C. (2013). Establishment of stably transfected cells constitutively expressing the full-length and truncated antigenic proteins of two genetically distinct mink astroviruses. PLoS ONE.

[37] Sellers H., Linneman E., Icard A.H., Mundt E. (2010). A purified recombinant baculovirus expressed capsid protein of a new astrovirus provides partial protection to runting-stunting syndrome in chickens. Vaccine.

[38] Johnson C., Hargest V., Cortez V., Meliopoulos V.A., Schultz-Cherry S. (2017). Astrovirus Pathogenesis. Viruses.

[39] Todd D., Wilkinson D.S., Jewhurst H.L., Wylie M., Gordon A.W., Adair B.M. (2009). A seroprevalence investigation of chicken astrovirus infections. Avian Pathol..

[40] Greenberg H.B., Matsui S.M. (1992). Astroviruses and caliciviruses: Emerging enteric pathogens. Infect. Agents Dis..

[41] De Benedictis P., Schultz-Cherry S., Burnham A., Cattoli G. (2011). Astrovirus infections in humans and animals—Molecular biology, genetic diversity, and interspecies transmissions. Infect. Genet. Evol..

[42] Donato C., Vijaykrishna D. (2017). The Broad Host Range and Genetic Diversity of Mammalian and Avian Astroviruses. Viruses.

[43] Smits S.L., van Leeuwen M., van der Eijk A.A., Fraaij P.L., Escher J.C., Simon J.H., Osterhaus A.D. (2010). Human astrovirus infection in a patient with new-onset celiac disease. J. Clin. Microbiol..

[44] Finkbeiner S.R., Li Y., Ruone S., Conrardy C., Gregoricus N., Toney D., Virgin H.W., Anderson L.J., Vinje J., Wang D. (2009). Identification of a novel astrovirus (astrovirus VA1) associated with an outbreak of acute gastroenteritis. J. Virol..

[45] Finkbeiner S.R., Le B.M., Holtz L.R., Storch G.A., Wang D. (2009). Detection of newly described astrovirus MLB1 in stool samples from children. Emerg. Infect. Dis..

[46] Marvin S.A. (2016). The Immune Response to Astrovirus Infection. Viruses.

[47] Bogdanoff W.A., Campos J., Perez E.I., Yin L., Alexander D.L., DuBois R.M. (2017). Structure of a Human Astrovirus Capsid-Antibody Complex and Mechanistic Insights into Virus Neutralization. J. Virol..

[48] Bogdanoff W.A., Perez E.I., Lopez T., Arias C.F., DuBois R.M. (2018). Structural Basis for Escape of Human Astrovirus from Antibody Neutralization: Broad Implications for Rational Vaccine Design. J. Virol..

[49] Gronemus J.Q., Hair P.S., Crawford K.B., Nyalwidhe J.O., Cunnion K.M., Krishna N.K. (2010). Potent inhibition of the classical pathway of complement by a novel C1q-binding peptide derived from the human astrovirus coat protein. Mol. Immunol..

[50] Bonaparte R.S., Hair P.S., Banthia D., Marshall D.M., Cunnion K.M., Krishna N.K. (2008). Human astrovirus coat protein inhibits serum complement activation via C1, the first component of the classical pathway. J. Virol..

[51] Tacket C.O., Sztein M.B., Losonsky G.A., Wasserman S.S., Estes M.K. (2003). Humoral, mucosal, and cellular immune responses to oral Norwalk virus-like particles in volunteers. Clin. Immunol..

[52] Wang S., Liu H., Zu X., Liu Y., Chen L., Zhu X., Zhang L., Zhou Z., Xiao G., Wang W. (2016). The ubiquitin-proteasome system is essential for the productive entry of Japanese encephalitis virus. Virology.

[53] Guix S., Perez-Bosque A., Miro L., Moreto M., Bosch A., Pinto R.M. (2015). Type I interferon response is delayed in human astrovirus infections. PLoS ONE.

[54] Marvin S.A., Huerta C.T., Sharp B., Freiden P., Cline T.D., Schultz-Cherry S. (2016). Type I Interferon Response Limits Astrovirus Replication and Protects against Increased Barrier Permeability In Vitro and In Vivo. J. Virol..

[55] Sen A., Sharma A., Greenberg H.B. (2017). Rotavirus degrades multiple type interferon receptors to inhibit IFN signaling and protects against mortality from endotoxin in suckling mice. J. Virol..

[56] Frias A.H., Jones R.M., Fifadara N.H., Vijay-Kumar M., Gewirtz A.T. (2012). Rotavirus-induced IFN-beta promotes anti-viral signaling and apoptosis that modulate viral replication in intestinal epithelial cells. Innate Immun..

[57] Koci M.D., Kelley L.A., Larsen D., Schultz-Cherry S. (2004). Astrovirus-induced synthesis of nitric oxide contributes to virus control during infection. J. Virol..

[58] Yokoyama C.C., Loh J., Zhao G., Stappenbeck T.S., Wang D., Huang H.V., Virgin H.W., Thackray L.B. (2012). Adaptive immunity restricts replication of novel murine astroviruses. J. Virol..

[59] Kapoor A., Li L., Victoria J., Oderinde B., Mason C., Pandey P., Zaidi S.Z., Delwart E. (2009). Multiple novel astrovirus species in human stool. J. Gen. Virol..

[60] Bass D.M., Upadhyayula U. (1997). Characterization of human serotype 1 astrovirus-neutralizing epitopes. J. Virol..

[61] Meyerhoff R.R., Nighot P.K., Ali R.A., Blikslager A.T., Koci M.D. (2012). Characterization of turkey inducible nitric oxide synthase and identification of its expression in the intestinal epithelium following astrovirus infection. Comp. Immunol. Microbiol. Infect. Dis..

[62] Koci M.D., Moser L.A., Kelley L.A., Larsen D., Brown C.C., Schultz-Cherry S. (2003). Astrovirus induces diarrhea in the absence of inflammation and cell death. J. Virol..

[63] Meliopoulos V.A., Marvin S.A., Freiden P., Moser L.A., Nighot P., Ali R., Blikslager A., Reddivari M., Heath R.J., Koci M.D. (2016). Oral Administration of Astrovirus Capsid Protein Is Sufficient to Induce Acute Diarrhea In Vivo. MBio.

[64] Woode G.N., Pohlenz J.F., Gourley N.E., Fagerland J.A. (1984). Astrovirus and Breda virus infections of dome cell epithelium of bovine ileum. J. Clin. Microbiol..

[65] Gray E.W., Angus K.W., Snodgrass D.R. (1980). Ultrastructure of the small intestine in astrovirus-infected lambs. J. Gen. Virol..

[66] Madhi S.A., Cutland C.L., Kuwanda L., Weinberg A., Hugo A., Jones S., Adrian P.V., van Niekerk N., Treurnicht F., Ortiz J.R. (2014). Influenza vaccination of pregnant women and protection of their infants. N. Engl. J. Med..

[67] Pulit-Penaloza J.A., Esser E.S., Vassilieva E.V., Lee J.W., Taherbhai M.T., Pollack B.P., Prausnitz M.R., Compans R.W., Skountzou I. (2014). A protective role of murine langerin(+) cells in immune responses to cutaneous vaccination with microneedle patches. Sci. Rep..

[68] Chiu C.H., Chu C., He C.C., Lin T.Y. (2006). Protection of neonatal mice from lethal enterovirus 71 infection by maternal immunization with attenuated Salmonella enterica serovar Typhimurium expressing VP1 of enterovirus 71. Microbes Infect..

[69] Kim Y.I., Song J.H., Kwon B.E., Kim H.N., Seo M.D., Park K., Lee S., Yeo S.G., Kweon M.N., Ko H.J. (2015). Pros and cons of VP1-specific maternal IgG for the protection of Enterovirus 71 infection. Vaccine.

[70] Munoz F.M. (2015). Respiratory syncytial virus in infants: Is maternal vaccination a realistic strategy?. Curr. Opin. Infect. Dis..

[71] Pan-Ngum W., Kinyanjui T., Kiti M., Taylor S., Toussaint J.F., Saralamba S., Van Effelterre T., Nokes D.J., White L.J. (2017). Predicting the relative impacts of maternal and neonatal respiratory syncytial virus (RSV) vaccine target product profiles: A consensus modelling approach. Vaccine.

[72] Eberhardt C.S., Blanchard-Rohner G., Lemaitre B., Combescure C., Othenin-Girard V., Chilin A., Petre J., Martinez de Tejada B., Siegrist C.A. (2017). Pertussis Antibody Transfer to Preterm Neonates After Second- Versus Third-Trimester Maternal Immunization. Clin. Infect. Dis..

[73] Eberhardt C.S., Blanchard-Rohner G., Lemaitre B., Boukrid M., Combescure C., Othenin-Girard V., Chilin A., Petre J., de Tejada B.M., Siegrist C.A. (2016). Maternal Immunization Earlier in Pregnancy Maximizes Antibody Transfer and Expected Infant Seropositivity Against Pertussis. Clin. Infect. Dis..

[74] Heath P.T. (2011). An update on vaccination against group B streptococcus. Expert Rev. Vaccines.

[75] Amirthalingam G., Andrews N., Campbell H., Ribeiro S., Kara E., Donegan K., Fry N.K., Miller E., Ramsay M. (2014). Effectiveness of maternal pertussis vaccination in England: An observational study. Lancet.

[76] Dabrera G., Amirthalingam G., Andrews N., Campbell H., Ribeiro S., Kara E., Fry N.K., Ramsay M. (2015). A case-control study to estimate the effectiveness of maternal pertussis vaccination in protecting newborn infants in England and Wales, 2012–2013. Clin. Infect. Dis..

[77] Janowski A.B., Bauer I.K., Holtz L.R., Wang D. (2017). Propagation of astrovirus VA1, a neurotropic human astrovirus, in cell culture. J. Virol..

[78] Gattoni A., Parlato A., Vangieri B., Bresciani M., Derna R. (2006). Interferon-gamma: Biologic functions and HCV therapy (type I/II) (1 of 2 parts). Clin. Ter..

[79] Molberg O., Nilsen E.M., Sollid L.M., Scott H., Brandtzaeg P., Thorsby E., Lundin K.E. (1998). CD4+ T cells with specific reactivity against astrovirus isolated from normal human small intestine. Gastroenterology.

[80] Souza M., Costantini V., Azevedo M.S., Saif L.J. (2007). A human norovirus-like particle vaccine adjuvanted with ISCOM or mLT induces cytokine and antibody responses and protection to the homologous GII.4 human norovirus in a gnotobiotic pig disease model. Vaccine.

[81] Guo L., Wang J., Zhou H., Si H., Wang M., Song J., Han B., Shu Y., Ren L., Qu J. (2008). Intranasal administration of a recombinant adenovirus expressing the norovirus capsid protein stimulates specific humoral, mucosal, and cellular immune responses in mice. Vaccine.

[82] Lopman B.A., Brown D.W., Koopmans M. (2002). Human caliciviruses in Europe. J. Clin. Virol..

