# Supplementary material for: Immunogenicity and Efficacy Evaluation of Subunit Astrovirus Vaccines
Source: Vaccines (Basel). 2019 Aug 2;7(3):79. doi: 10.3390/vaccines7030079 (PMC6789684; doi:10.3390/vaccines7030079)
Supplement: Supplementary file 1 [file vaccines-07-00079-s001.pdf]

(A)

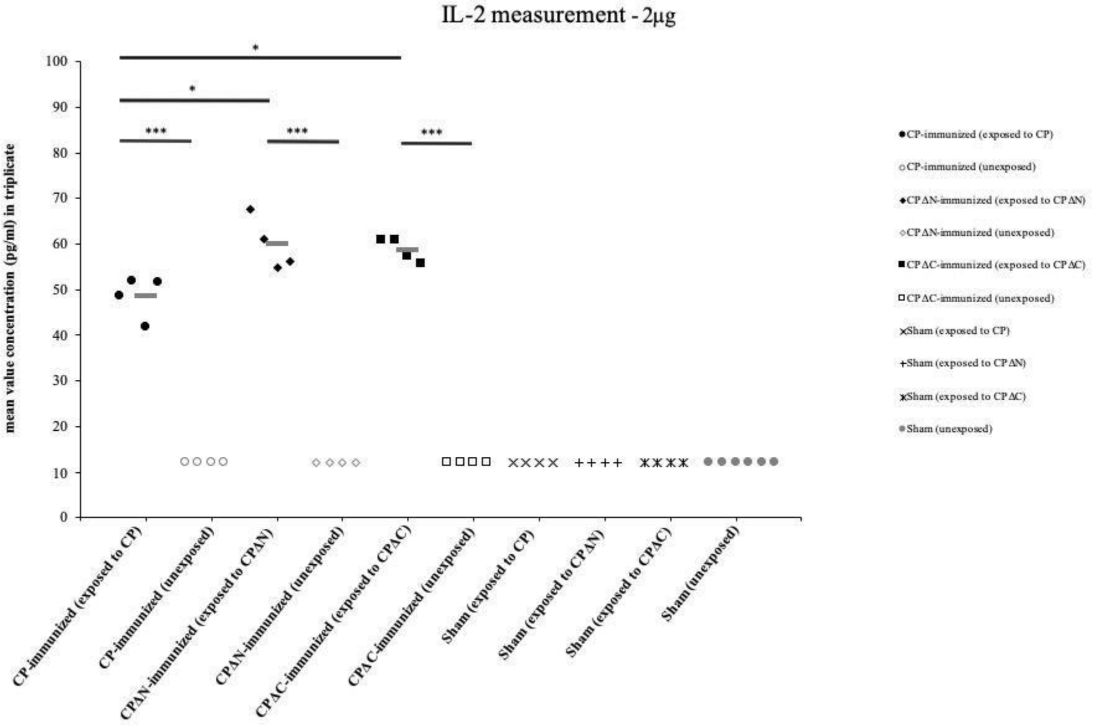
IL-2 measurement - 2µg
mean value concentration (pg/ml) in triplicate
100
90
80
70
60
50
40
30
20
10
0
*
*
***
***
***
CP-immunized (exposed to CP)
CP-immunized (unexposed)
CPΔN-immunized (exposed to CPΔN)
CPΔN-immunized (unexposed)
CPΔC-immunized (exposed to CPΔC)
CPΔC-immunized (unexposed)
Sham (exposed to CP)
Sham (exposed to CPΔN)
Sham (exposed to CPΔC)
Sham (unexposed)

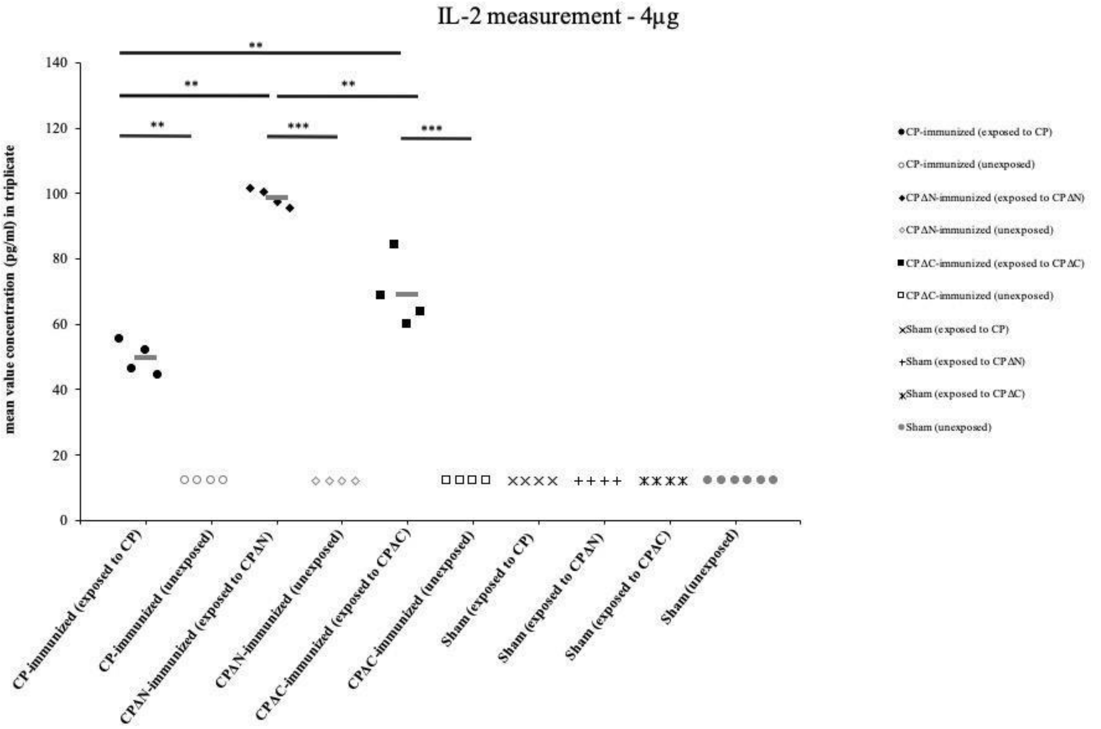
IL-2 measurement - 4µg
mean value concentration (pg/ml) in triplicate
140
120
100
80
60
40
20
0
**
**
**
**
***
***
CP-immunized (exposed to CP)
CP-immunized (unexposed)
CPΔN-immunized (exposed to CPΔN)
CPΔN-immunized (unexposed)
CPΔC-immunized (exposed to CPΔC)
CPΔC-immunized (unexposed)
Sham (exposed to CP)
Sham (exposed to CPΔN)
Sham (exposed to CPΔC)
Sham (unexposed)

(B)

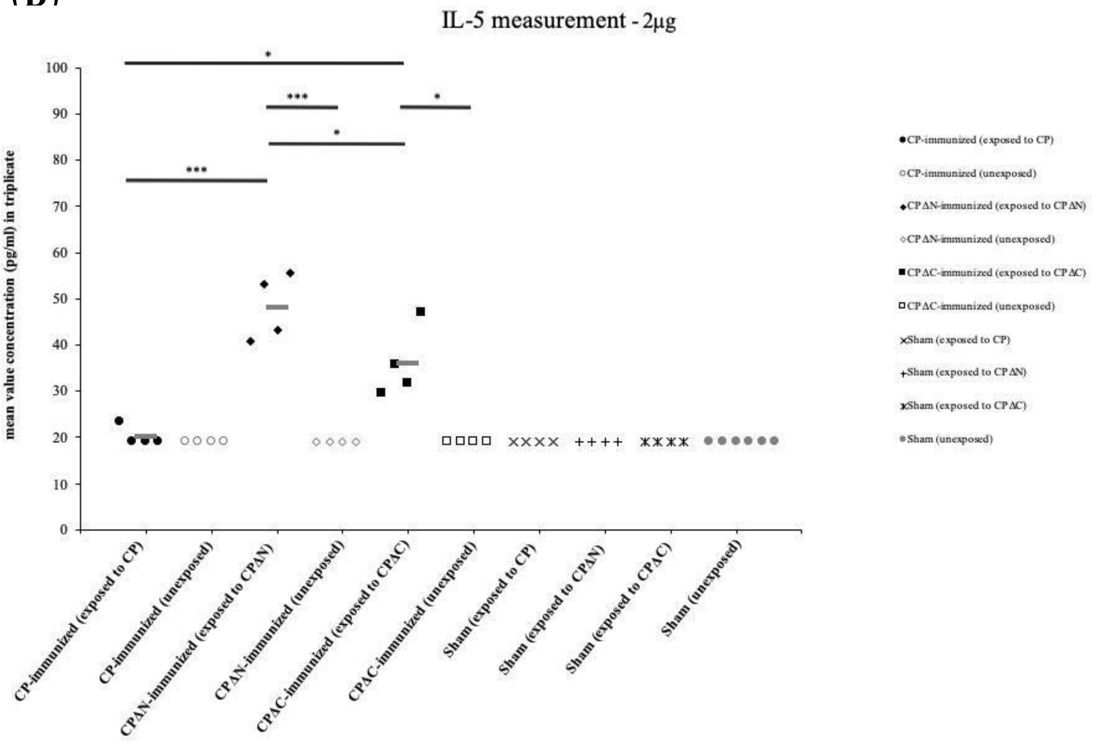
IL-5 measurement - 2μg
mean value concentration (pg/ml) in triplicate
*
***
*
*
***
CP-immunized (exposed to CP)
CP-immunized (unexposed)
CPΔN-immunized (exposed to CPΔN)
CPΔN-immunized (unexposed)
CPΔC-immunized (exposed to CPΔC)
CPΔC-immunized (unexposed)
Sham (exposed to CP)
Sham (exposed to CPΔN)
Sham (exposed to CPΔC)
Sham (unexposed)

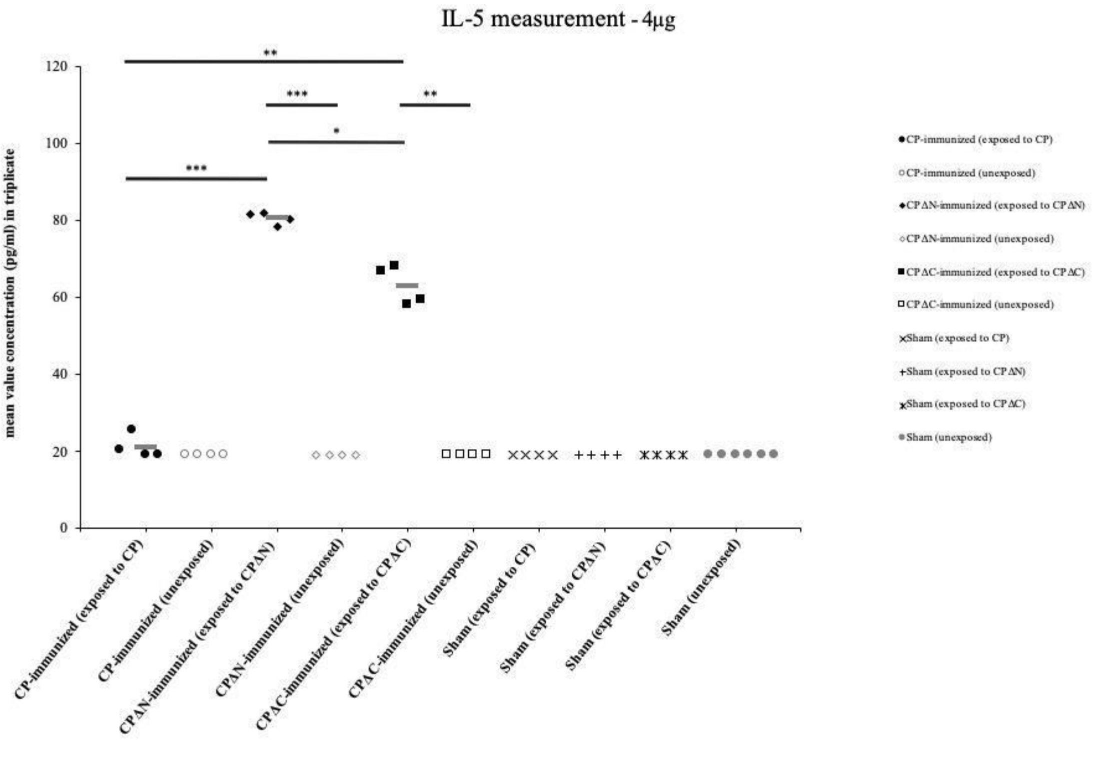
IL-5 measurement - 4μg
mean value concentration (pg/ml) in triplicate
**
***
**
*
***
CP-immunized (exposed to CP)
CP-immunized (unexposed)
CPΔN-immunized (exposed to CPΔN)
CPΔN-immunized (unexposed)
CPΔC-immunized (exposed to CPΔC)
CPΔC-immunized (unexposed)
Sham (exposed to CP)
Sham (exposed to CPΔN)
Sham (exposed to CPΔC)
Sham (unexposed)

(C)

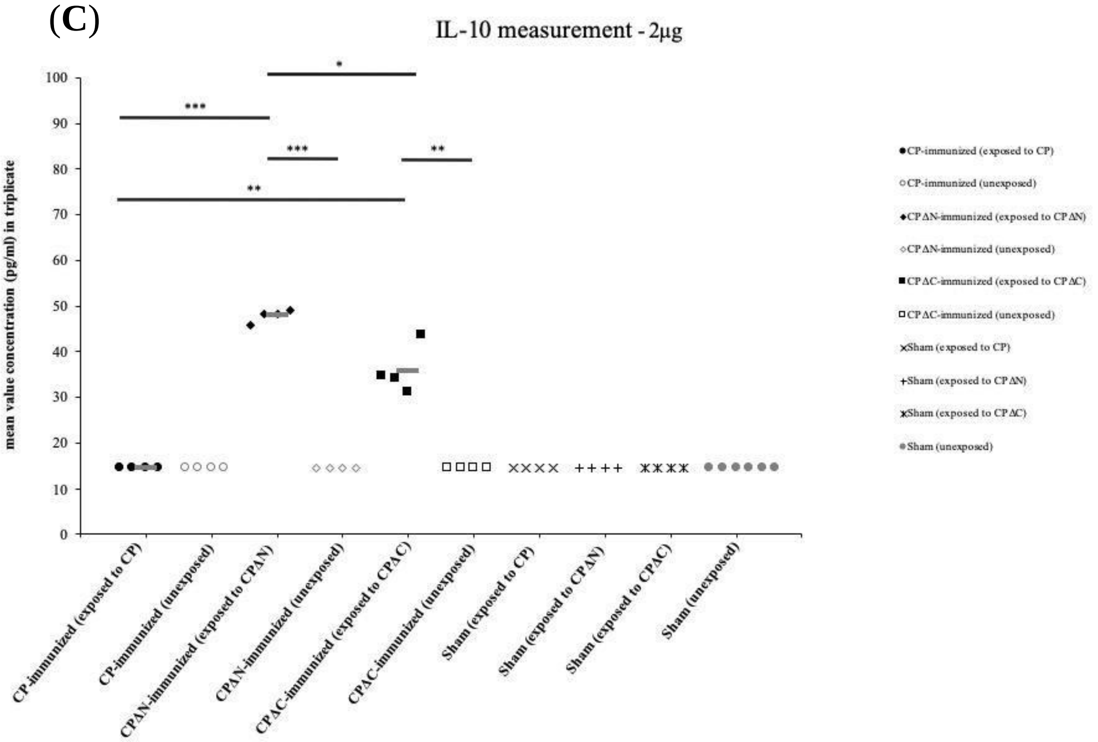
IL-10 measurement - 2μg
mean value concentration (pg/ml) in triplicate
100
90
80
70
60
50
40
30
20
10
0
***
*
***
**
**
CP-immunized (exposed to CP)
CP-immunized (unexposed)
CPΔN-immunized (exposed to CPΔN)
CPΔN-immunized (unexposed)
CPΔC-immunized (exposed to CPΔC)
CPΔC-immunized (unexposed)
Sham (exposed to CP)
Sham (exposed to CPΔN)
Sham (exposed to CPΔC)
Sham (unexposed)

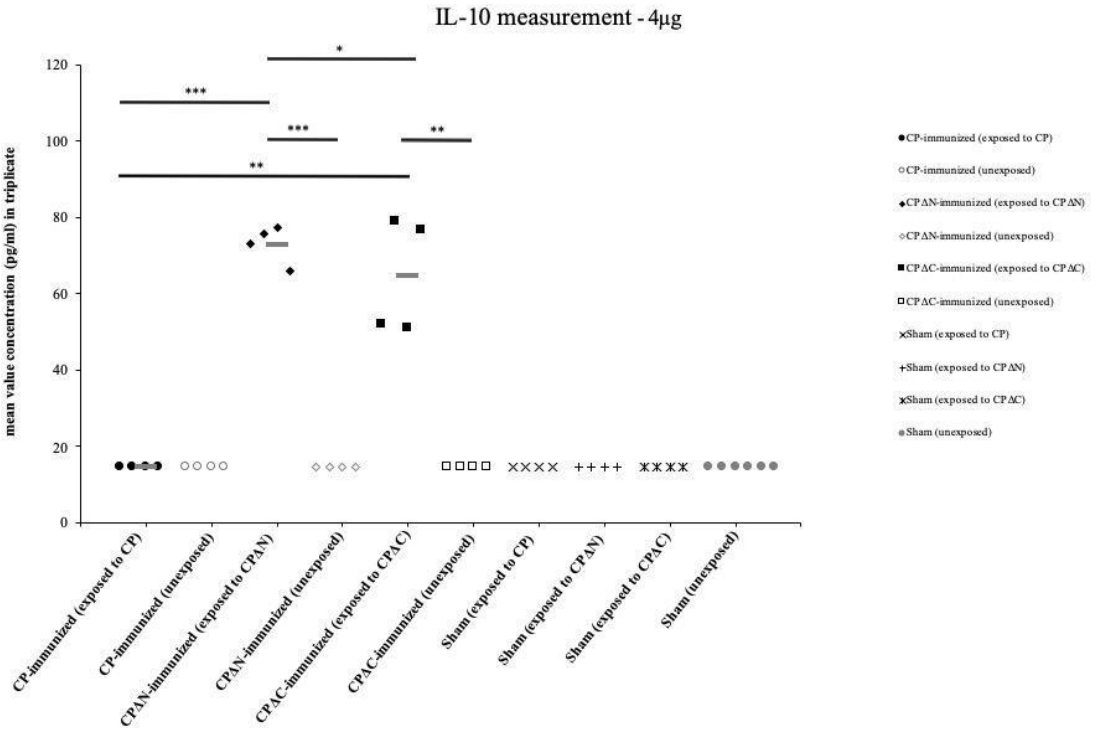
IL-10 measurement - 4μg
mean value concentration (pg/ml) in triplicate
120
100
80
60
40
20
0
***
*
***
**
**
CP-immunized (exposed to CP)
CP-immunized (unexposed)
CPΔN-immunized (exposed to CPΔN)
CPΔN-immunized (unexposed)
CPΔC-immunized (exposed to CPΔC)
CPΔC-immunized (unexposed)
Sham (exposed to CP)
Sham (exposed to CPΔN)
Sham (exposed to CPΔC)
Sham (unexposed)

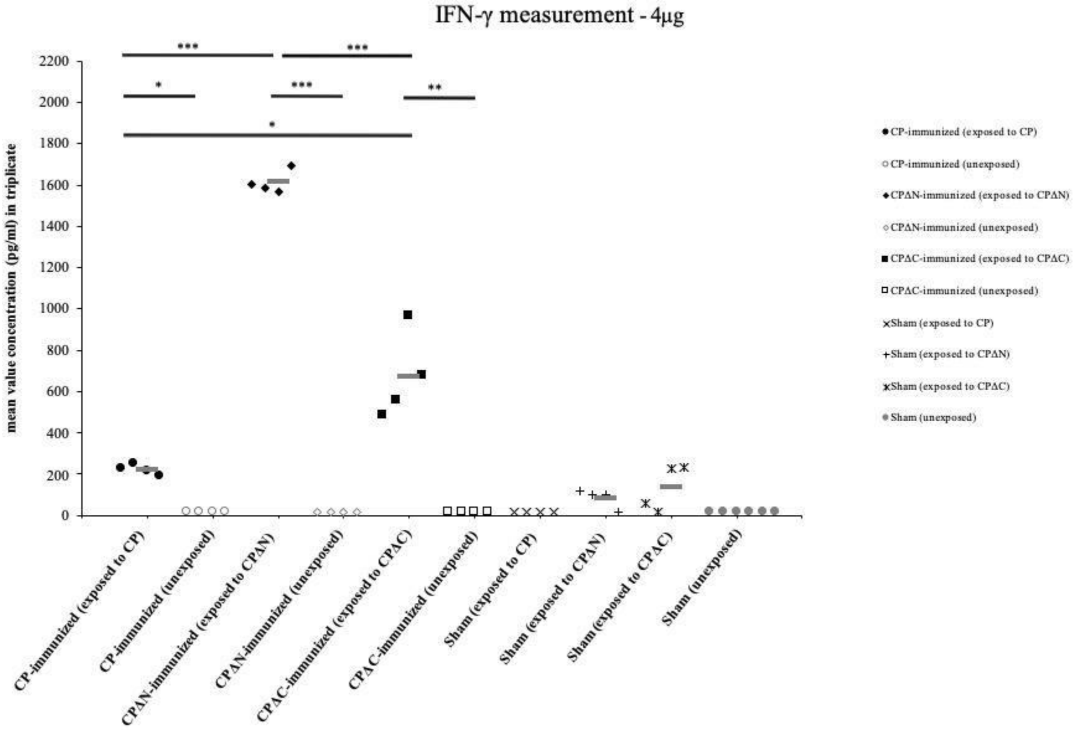

(D)

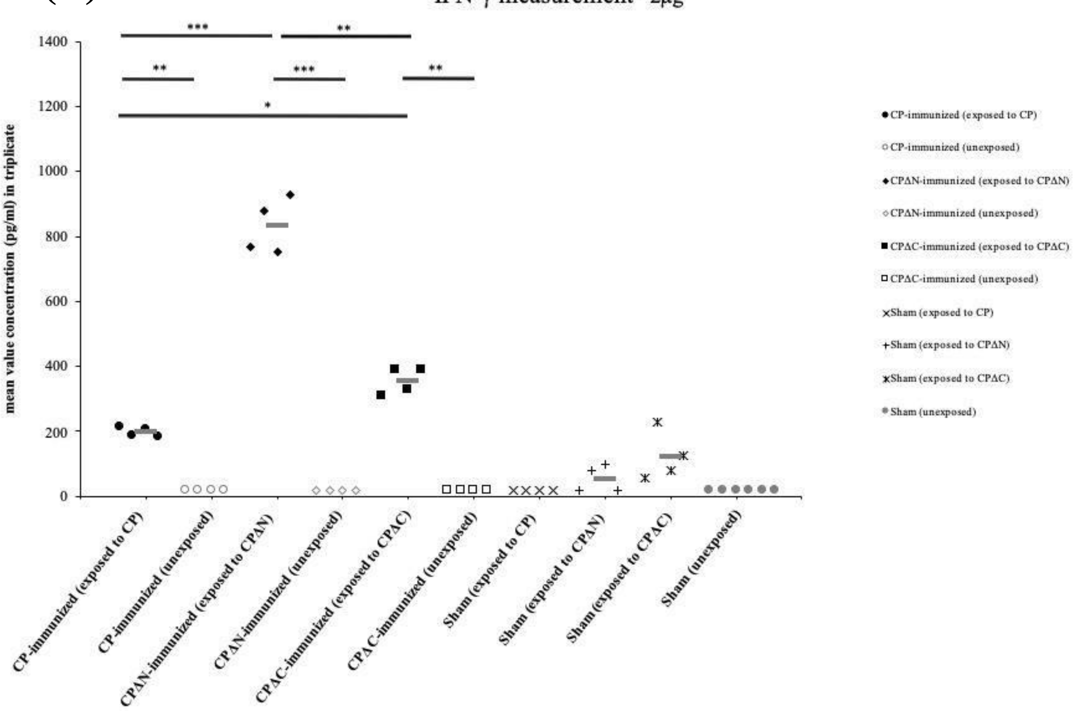

Supplementary Figure 1. Analyses of T cell-mediated immune response to the corresponding MAstV CPs. The MAstV vaccine candidates (5 μg/mouse) combined with an equal volume of AbISCO-100 adjuvant (10 μg/mouse) were injected to NMRI mice (*n* =8 per group, 4 weeks old) twice with a three week interval. Mice injected with pDual-GC-vector transfected cell lysate combined with adjuvant was also analyzed as Sham (*n* = 8). Three weeks after second immunization, mice splenocytes were harvested from each group of mice: CP-immunized mice, CPΔN-immunized mice, and CPΔC- immunized mice. Mice injected with pDual-GC-vector transfected cell lysate combined with adjuvant was also analyzed as Sham (*n* = 8). The splenocytes of four mice per group were then extracted,

washed, and cultivated ex vivo (2×10^5 cells/well) and stimulated by exposing to corresponding MAstV

CPs as described in Materials and Methods, and the culture supernatants from stimulated splenocytes were collected and assessed for secreted cytokines (**A**) IL-2, (**B**) IL-5, (**C**) IL-10, and (**D**) IFN-γ by using a mouse Th1/Th2 Cytokine Cytometric 6-plex Array Bead kit (Invitrogen), Luminex® 100/200™ System and xPONENT® software (Luminex Corporation, Austin, Texas). The splenocytes of Sham group (*n* = 8) were also independently exposed to each of the three MAstV CPs. The data are mean COD readings of duplicate experiments which is illustrated in dot plot for each measurement. Asterisks indicate level of significant difference between mean value of either stimulated or unstimulated cells using student t-test (two-tailed distribution, two-sample unequal variance, Heteroscedastic); * $p < 0.05$, ** $p < 0.01$, and *** $p < 0.001$. Gray bars represent mean value for each group.

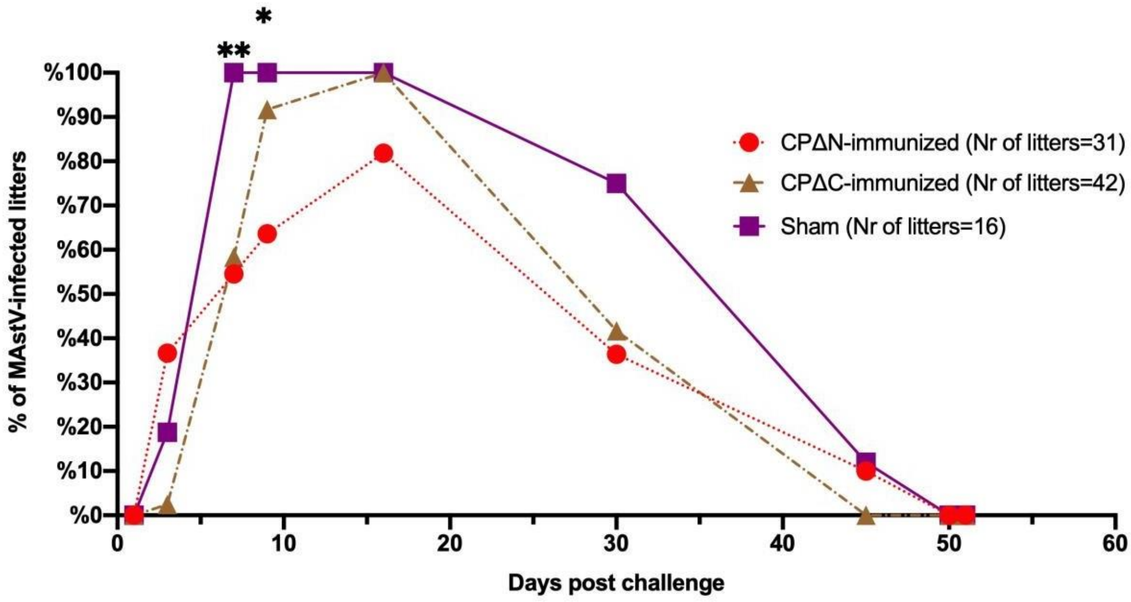

Supplementary Figure 2. Graph of results of real-time PCR and clinical sign observation in experiment of litters mink trial. After birth, the one-day newborn litters (*n* = 89) of immunized and Sham adult moms (*n* = 17) were challenged with 10^7/ml MAstV copies. As a follow-up, the fecal samples (*n* = 414) of their newborn litters on test days 1, 3, 5, 7, 9, 16, 30, 45, 50, and 51 post challenge were tested using a real-time PCR for detecting AstV shedding status. The clinical signs of litters were also monitored and recorded by the author veterinarians every other day. The ratio of PCR-positive litters to total sampled litters on each sampling occasion during challenge experiment (51 days) was calculated and illustrated as percentage (%) in a graph. Using a real-time PCR detecting the conserved NS gene of MAstV (Ct value is 20, Threshold = 0.02), positive samples were detected. Asterisks show significant decline of virus shedding among litters of either both CPΔN- and CPΔC- (**) or just CPΔN- (*) immunized adult mom groups compared to those of Sham group using the Log-rank test ($p < 0.05$).
